# A Posture-Constrained Infrared Thermography Framework for Dairy Cow Mastitis Detection with DAT-YOLO26

**DOI:** 10.3390/ani16142220

**Published:** 2026-07-17

**Authors:** Gegerihu Bao, Xiao Jin, Jing Gao, Xintong Li, Zhipeng Han, Jian Song

**Affiliations:** 1 College of Computer and Information Engineering, Inner Mongolia Agricultural University, Erdos East Street No. 29, Hohhot 010011, China; gegerihu@gmail.com; 2 School of Computer and Information Technology, Hohhot Minzu College, North Tongdao Road No. 56, Hohhot 010051, China; 3 Inner Mongolia Autonomous Region Key Laboratory of Big Data Research and Application for Agriculture and Animal Husbandry, Zhaowuda Road No. 306, Hohhot 010018, China; 4 College of Animal Science, Inner Mongolia Agricultural University, Zhaowuda Road No. 306, Hohhot 010018, China; yaojinxiao@imau.edu.cn (X.J.); lixintongaa@163.com (X.L.); hzp4639@163.com (Z.H.); 18031478235@163.com (J.S.); 5 Inner Mongolia Autonomous Region Bureau of Government Services and Data Management, Chilechuan Street No. 1, Hohhot 010091, China

**Keywords:** infrared thermography, dairy cow mastitis detection, posture estimation, instance segmentation, diagnostic accuracy

## Abstract

Bovine mastitis, a prevalent and economically detrimental inflammatory disease in dairy cattle, leads to substantial economic losses due to reduced milk yield, impaired milk quality, and increased veterinary costs. Infrared thermography (IRT) provides an efficient, non-invasive approach for early mastitis detection by leveraging computer vision to analyze temperature differences between ocular and udder regions. However, in practical farm environments, arbitrary head movements, particularly horizontal rotation, alter sensor-to-object distance, introducing systematic biases in thermal measurements and degrading diagnostic reliability. To address this limitation, a two-stage detection framework is proposed under a lateral infrared imaging configuration, in which the camera is positioned perpendicular to the walking direction of cows. First, a posture classification module based on pose estimation is employed to identify cows with consistent lateral orientation and minimal head rotation, thereby maintaining stable sensor-to-object distance between the ocular and udder regions. Second, instance segmentation is applied to posture-constrained thermal images to achieve accurate regional temperature extraction and quantitative analysis. In addition, an improved YOLO26 model integrated with a Deformable Attention Transformer (DAT-YOLO26) is developed to enhance feature representation capability and detection performance under variable imaging conditions. On an independent 40-cow blind diagnostic cohort (20 healthy, 20 mastitis-positive), the proposed method achieved an accuracy of 87.5%, an F1-score of 87.18%, a sensitivity of 85%, and a specificity of 90%. These results, while constrained by the size of the test cohort, suggest that enforcing posture consistency improves IRT-based diagnostic performance under the present acquisition conditions. We emphasize, however, that these findings are based on a single-farm, single-season pilot cohort of 40 cows; multi-farm and multi-season validation are required before broader deployment claims can be made.

## Simple Summary

Mastitis, an inflammation of the udder, is one of the most common and costly diseases affecting dairy cows. It lowers both the amount and the quality of the milk a cow produces and can harm the animal’s health and comfort. Detecting the disease early—particularly the hidden, subclinical form that shows no obvious outward signs—helps farmers respond sooner and reduces both animal suffering and economic loss. Infrared thermography cameras can measure the surface temperature of a cow’s body without any physical contact, and an inflamed udder tends to be slightly warmer than a healthy one. However, when a cow turns its head as it walks past the camera, the changing distance between the animal and the sensor distorts these temperature readings and can lead to mistaken results. In this study, we developed a computer system that first checks each cow’s body posture and keeps only the images captured when the animal faces a consistent direction, and then measures the temperature of the eye and udder regions to flag possible cases of mastitis. Tested on a small group of cows at a single farm, the approach showed promising accuracy, although testing across more farms is needed before it could be used widely.

## 1. Introduction

According to data obtained from the May 2026 customized online query of the Production, Supply and Distribution (PSD) database maintained by the Foreign Agricultural Service of the United States Department of Agriculture (USDA-FAS) [1], the stock of dairy cows in China has reached approximately 12.5 million head, placing China among the countries with the largest dairy cow herds across the globe. Bovine mastitis is still one of the most economically destructive and biologically intricate diseases affecting dairy cows globally. This disease manifests as mammary gland inflammation, which is predominantly induced by bacterial infections, and consequently causes substantial declines in both milk production and milk quality [2].

Mastitis is commonly categorized into clinical mastitis and subclinical mastitis. Current academic research mainly focuses on optimizing diagnostic strategies, innovating novel detection and therapeutic technologies (including nanotechnology and probiotic therapy), exploring antimicrobial resistance mechanisms, as well as refining prevention strategies via genetic breeding selection and standardized farm hygiene management. Traditional diagnostic methods for mastitis have long adopted contact-based or invasive detection means. In the screening of subclinical mastitis, somatic cell count (SCC) is the most widely used herd-level indicator, which reflects the infiltration of leukocytes into mammary tissues triggered by infectious lesions. Other mainstream detection approaches cover the California Mastitis Test (CMT) and milk electrical conductivity (EC) detection. The CMT can provide on-site qualitative evaluation of somatic cell counts in dairy farms, while EC detection judges disease status by monitoring variations in ionic concentration within milk.

Although SCC can effectively characterize the overall health condition of cow udders, it fails to achieve the accurate identification of specific pathogenic bacteria. Visual observation and manual palpation feature simple operation and low application costs, yet they suffer from insufficient detection sensitivity and specificity, especially in the identification of subclinical mastitis. The CMT is easy to popularize and apply in farm environments, but it has relatively low detection accuracy and cannot obtain pathogen-specific detection results. Electrical conductivity detection supports fast, non-invasive and real-time continuous monitoring; nevertheless, its test results are susceptible to multiple external interference factors such as milk composition differences and different lactation stages, which may further trigger false-positive diagnosis results [3].

All the above conventional detection approaches are labor-intensive, dependent on dedicated chemical reagents and professional testing equipment, and difficult to popularize and apply in modern large-scale and high-efficiency dairy breeding industries. Therefore, it is an urgent research direction in this field to develop non-invasive, intelligent, automatic, and high-precision mastitis diagnostic systems.

Infrared thermography (IRT) has emerged as an advanced non-invasive diagnostic technique for bovine mastitis, offering prominent advantages in operational efficiency and real-time monitoring [4,5,6,7,8]. Metzner et al. [9] used IRT to evaluate udder surface temperature changes in cows following experimental Escherichia coli mastitis induction in the right hind quarter. Thermal images were acquired every 2 h from 24 h before inoculation to 24 h after inoculation, and both maximum and average udder temperatures were analyzed. All cows developed clinical mastitis, accompanied by significantly elevated temperature differences. The results confirmed that IRT allows effective mastitis detection when the imaging interval does not exceed 2 h. Sathiyabarathi et al. [10,11] proposed IRT as a non-invasive and rapid diagnostic tool for bovine mastitis, using ocular surface temperature ($T_{\mathrm{eye}}$) as a stable core body temperature reference to quantify variations in udder skin surface temperature ($T_{\mathrm{udder}}$). In a study involving Bos indicus (Deoni) cows [10], IRT detected a 1.51 °C increase in $T_{\mathrm{udder}}$ in subclinically affected quarters relative to $T_{\mathrm{eye}}$ (36.10 ± 0.08 °C), with strong correlations with SCC (R^2^ > 0.95) and EC. The reliability of $T_{\mathrm{eye}}$ arises from its physiological stability, as the ophthalmic artery—a branch of the internal carotid artery—supplies blood to the eye, making ocular temperature less susceptible to external interference such as hair coverage or ambient temperature. Notably, ocular IR temperature has been reported to exhibit greater consistency than that of any other anatomical region [10].

Another study on Holstein Friesian crossbred cows also validated the efficacy of IRT, revealing $T_{\mathrm{udder}}$ increments of 0.72 °C in subclinical mastitis and 1.05 °C in clinical mastitis compared with healthy quarters, with $T_{\mathrm{eye}}$ (37.23 ± 0.08 °C) used as a standardized baseline [11]. The non-invasive protocol supports on-site real-time screening, and ROC analysis established a $T_{\mathrm{udder}}$ threshold of  >37.61 °C, yielding 71.51–100% sensitivity for early subclinical mastitis detection. By normalizing $T_{\mathrm{udder}}$ against $T_{\mathrm{eye}}$, these studies demonstrate the dual strengths of IRT: physiological validity based on the close association between $T_{\mathrm{eye}}$ and core body temperature, and practical feasibility through non-invasive high-resolution thermal imaging that captures inflammatory $T_{\mathrm{udder}}$ elevations. These findings establish a reliable theoretical foundation for applying IRT in udder health monitoring of dairy cows.

In early investigations, researchers manually captured infrared images and performed temperature comparisons, resulting in relatively low analytical efficiency. With the maturation of computer vision, current IRT-based mastitis detection research can be broadly grouped into two complementary paradigms. The first, which we refer to as the eye–udder differential-temperature paradigm, uses deep networks to localize the ocular and mammary regions, extracts the corresponding surface temperatures $T_{\mathrm{eye}}$ and $T_{\mathrm{udder}}$, and renders a diagnosis through an explicit, physiologically grounded temperature-difference indicator $\Delta T=T_{\mathrm{udder}}-T_{\mathrm{eye}}$ [12,13,14,15,16]. This paradigm preserves intermediate quantitative readings—the temperatures themselves and their differentials—which can be inspected, audited, and cross-checked against established physiological reference values by farmers and veterinarians. The second paradigm reformulates mastitis detection as an end-to-end image-classification problem: a deep network is trained to map a thermal image (or a fused set of thermal-image features) directly to a disease label, with no explicit thermal value exposed to the user. The two paradigms trade off interpretability against end-to-end optimizability, and address partially non-overlapping sources of error [17,18,19,20,21].

In recent years, the development of advanced automated methods to achieve more accurate and efficient identification of ocular and udder regions has emerged as an active research challenge in dairy cow thermal imaging analysis [14,15,16]. Deep learning, with its capacity to automatically extract high-dimensional features from complex data, provides a powerful solution to the aforementioned limitations. Convolutional neural networks (CNNs) [22,23,24,25] have demonstrated superior performance in localizing anatomical regions such as the eye and udder, which is critical for accurate temperature difference calculation. By computing the ΔT, the influences of ambient temperature fluctuations and individual physiological variations can be effectively alleviated.

Representative studies of the first paradigm have therefore focused on improving the accuracy and efficiency of ocular and udder region localization prior to differential-temperature analysis. Wang et al. [12] proposed an improved YOLO v3-tiny object detection algorithm to further improve the localization accuracy and efficiency of the eye and udder regions, achieving a mastitis detection accuracy of 77.3%. Zhang et al. [13] developed the CLE-UNet algorithm, which integrates an attention mechanism and a novel centroid loss function to enhance the accuracy of mastitis detection through thermal infrared imaging. Wang et al. [15] promoted automated mastitis detection by emphasizing robust target localization and multi-indicator diagnostic criteria. By using YOLOv5 to extract eye and udder temperature information and combining bilateral $T_{\mathrm{udder}}$ differences with $T_{\mathrm{eye}}$ differences, the proposed method reduces external interference and yields a mastitis detection accuracy of 87.62%.

The second paradigm forgoes explicit temperature extraction and instead trains a deep network to classify thermal imagery directly. Chu et al. [18] combined a YOLOv7 detector for ocular and udder localization with a CenterNet keypoint network to recover udder geometric size; rather than reporting ΔT, the temperature and size descriptors were concatenated into a feature vector and classified by a wrapper-based support vector machine, yielding 88.61% accuracy on 196 dairy cows. Chu et al. [19] subsequently integrated object segmentation with blind motion deblurring and fed bilateral udder thermograms into a multi-scale spatial-and-channel squeeze-and-excitation DenseNet-201 (MS-scSE-DenseNet-201), training the network to discriminate three mastitis severity levels (negative, subclinical, clinical) directly from imagery, reaching 90.18% accuracy on 5000 thermograms collected from 802 cows. Along the same direction, Li, Chu et al. [20] proposed a multi-feature image-layer fusion strategy that aggregates complementary cues across intermediate CNN layers to support end-to-end mastitis classification. To address the small-dataset regime that is common in farm-level studies, Silva et al. [17] adopted a complementary strategy based on sequential cross-domain transfer learning: a ResNet50 backbone was first pre-trained on ImageNet, fine-tuned on human breast thermography images (MammoTherm), and finally adapted to a small cohort of 165 udder thermograms from 55 cows; with Bayesian hyperparameter optimization, the system reached 92.1% binary classification accuracy against the California Mastitis Test as the reference standard. By optimizing the entire pipeline toward the disease-label objective, this family of methods achieves strong empirical performance and benefits from generic representation learning and end-to-end optimizability; however, because the diagnostic decision is not anchored to a reported temperature value, the intermediate physiological quantities that veterinarians traditionally rely on are not exposed. Sharvanthika et al. [21] adopted visible-light udder images and a ResNet50-ViT hybrid model for binary mastitis classification. They enlarged 200 original images to 1150 samples via data augmentation, with the final classification accuracy of 94.67%. Differing from field datasets with definite individual cow numbers, this study only presented image statistics, and the number of independent dairy cows was not stated in the original text.

The accuracy of infrared thermography in field applications is governed not only by the intrinsic properties of the imaging sensor but also by a number of external factors, among which the sensor-to-object distance has been repeatedly identified as one of the principal sources of systematic error [26,27,28]. Mazdeyasna et al. [27] systematically reviewed best practices for body-temperature measurement with IRT and attributed distance-induced bias to the combined effects of atmospheric attenuation, defocus, and changes in the effective target solid angle relative to the detector’s instantaneous field of view. Zhang et al. [28] further demonstrated experimentally that even modest variations in sensor-to-object distance produce non-negligible drift in the apparent temperature reported by an infrared thermal imager, and proposed a distance-dependent compensation scheme to mitigate this effect. These findings indicate that, even under otherwise stable barn conditions (controlled ambient temperature, humidity, and emissivity), uncontrolled changes in sensor-to-object distance can directly compromise the reliability of IRT-based diagnosis.

In the lateral imaging configuration adopted in this study—with the camera fixed perpendicular to the cattle passage—the most prominent source of such distance variation is the cow’s own head movement. When the cow rotates its head toward or away from the sensor, the sensor-to-object distance for the ocular region changes appreciably, while the sensor-to-object distance for the udder region remains largely unchanged. Within a differential diagnostic framework that relies on $\Delta T=T_{\mathrm{udder}}-T_{\mathrm{eye}}$, this asymmetric distance change biases $T_{\mathrm{eye}}$ relative to $T_{\mathrm{udder}}$ and distorts ΔT, thereby compromising the reliability of subclinical mastitis detection.

**Figure 1:**
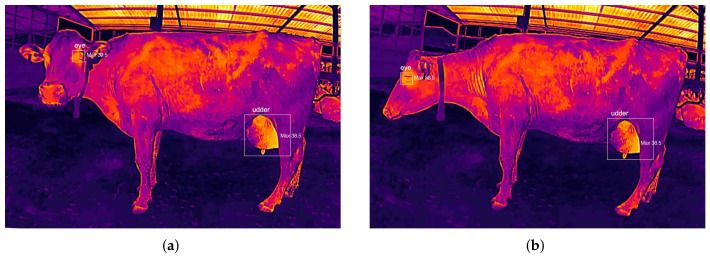
The maximum $T_{\mathrm{eye}}$ and $T_{\mathrm{udder}}$ for an identical dairy cow are compared under distinct postural states. In subplot (**a**), the max $T_{\mathrm{eye}}$ is measured as 39.5 °C when the cow turns its head toward the infrared thermal camera. In subplot (**b**), the eye temperature decreases to 38.5 °C under the lateral forward-moving posture with the head facing forward vertically.

This effect was directly observed during data collection. As illustrated in Figure 1, markedly different thermal readings were recorded for the same individual at the same anatomical site under distinct head postures. In the forward-moving posture (Figure 1b), the maximum $T_{\mathrm{eye}}$ and $T_{\mathrm{udder}}$ were both 38.5 °C; when the head was rotated toward the camera (Figure 1a), $T_{\mathrm{eye}}$ rose to 39.5 °C while $T_{\mathrm{udder}}$ remained at 38.5 °C. This 1.0 °C posture-induced shift in $T_{\mathrm{eye}}$ alone is comparable in magnitude to the inflammation-driven ΔT elevations reported in the literature for subclinical mastitis (∼0.7–1.5 °C [10,11]), making head posture a critical confounding factor that must be controlled prior to any thermal differential analysis. This observation suggests that head posture may introduce a clinically meaningful bias into ΔT estimation and therefore motivates the posture-filtering strategy investigated in this study.

To minimize measurement errors caused by varying cow orientations, we developed an improved YOLO26 [29,30] pose estimation algorithm to enable sequential pose-guided thermal analysis. The diagnostic pipeline is executed in two primary stages: The system first performs real-time pose recognition to filter the cow’s posture. A strict vertical alignment criterion is enforced, where the algorithm identifies instances where the cow’s head and longitudinal body axis form a vertical line relative to the sensor’s focal plane. This alignment reduces perspective distortion and ensures that the subsequent thermal feature extraction is performed under standardized anatomical conditions.

Once the optimal pose is locked, a secondary YOLO26-seg module performs concurrent instance segmentation on two critical Thermal Interest Areas: the eye region and the udder region. The segmentation masks ($M_{eye},M_{udder}$) allow for the precise extraction of pixel-level temperature $T_{\mathrm{eye}}$ and $T_{\mathrm{udder}}$ data. By comparing these regional temperatures, the system mitigates environmental noise and provides a more accurate assessment of the cow’s physiological condition.

We propose an improved YOLO26 model based on Deformable Attention Transformer (DAT-YOLO26) [31], to address challenges associated with complex backgrounds in real-time visual detection tasks. By integrating the deformable attention mechanism of DAT into the backbone of YOLO, the model can adaptively focus on key target regions and dynamically sample informative features, while maintaining linear computational complexity. Furthermore, we optimize multi-scale feature fusion and the detection head to enhance the representation of occluded objects. Experimental results demonstrate that DAT-YOLO26 achieves significant improvements in mAP and recall compared with the baseline YOLO26 model.

Within this taxonomy, the present work belongs to the differential- temperature paradigm: our pipeline reports explicit $T_{\mathrm{eye}}$, $T_{\mathrm{udder}}$ and ΔT values together with an interpretable threshold criterion, and is therefore directly compatible with the established physiological literature on IRT-based mastitis screening. We deliberately retain this paradigm because temperature-level traceability is valuable for veterinary auditing, threshold re-calibration across farms or seasons, and integration with existing herd-management workflows. Within the paradigm, however, prior studies have largely treated the eye as a stable thermal baseline without controlling for its sensitivity to sensor-to-object distance under spontaneous head rotation. The present work specifically targets this gap: a posture-classification gateway (DAT-YOLO26-pose with an SVM classifier) is placed upstream of segmentation and temperature extraction to ensure that $T_{\mathrm{eye}}$ is measured under a standardized geometry, so that the resulting ΔT is physically meaningful. The proposed framework is therefore complementary to the image- classification approach of Chu et al. [18,19,20]: rather than absorbing geometric variability into a learned representation at the cost of explicit temperature reporting, we remove a previously-overlooked geometric bias mechanistically and preserve the interpretability of the differential-temperature decision rule.

**Figure 2:**
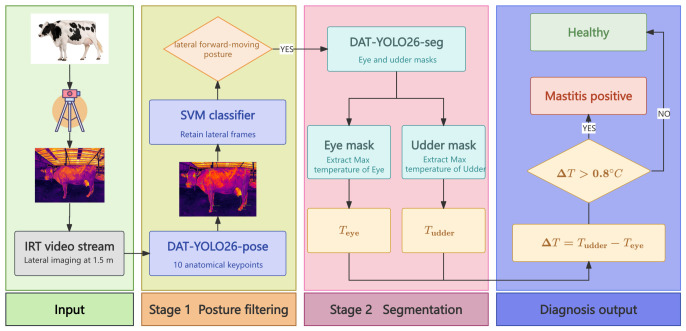
Overall framework of the proposed posture-constrained mastitis detection pipeline. Stage 1 filters frames by lateral forward-moving posture using DAT-YOLO26-pose and an SVM classifier. Stage 2 generates eye and udder masks via DAT-YOLO26-seg, from which $T_{\mathrm{eye}}$ and $T_{\mathrm{udder}}$ are extracted. Diagnosis output applies the differential temperature criterion ΔT>0.8 °C to issue the binary diagnostic decision.

The overall architecture of the proposed posture-constrained mastitis detection pipeline is illustrated in Figure 2. The workflow is formally described as follows: The model initially detects the bovine skeletal structure with DAT-YOLO26-pose. A posture filter is applied to select frames in which the head and torso exhibit vertical collinearity. This orientation-aware filtering minimizes the influence of viewing angle variations on infrared radiation measurements.

Following pose validation, the instance segmentation sub-model DAT-YOLO26-seg generates high-precision masks for the ocular and mammary regions. Let $S_{i}$ represent the segmented region for class i∈{eye,udder}. The maximum temperature $T_{\max,i}$ within the target region is calculated as Equation (1):

(1) $$T_{\max,i}={\mathrm{Max}}_{\left(x,y\right)\in S_{i}}I_{thermal}\left(x,y\right)$$

where $I_{thermal}$ is the mapped thermal radiance matrix.

A comparative analysis between $T_{\mathrm{eye}}$ and $T_{\mathrm{udder}}$ is conducted to establish a baseline for thermal homeostasis monitoring, thereby reducing the error margin compared with single-point measurements.

## 2. Materials and Methods

### 2.1. Data Acquisition

#### 2.1.1. Experimental Setup and Instrumentation

**Figure 3:**
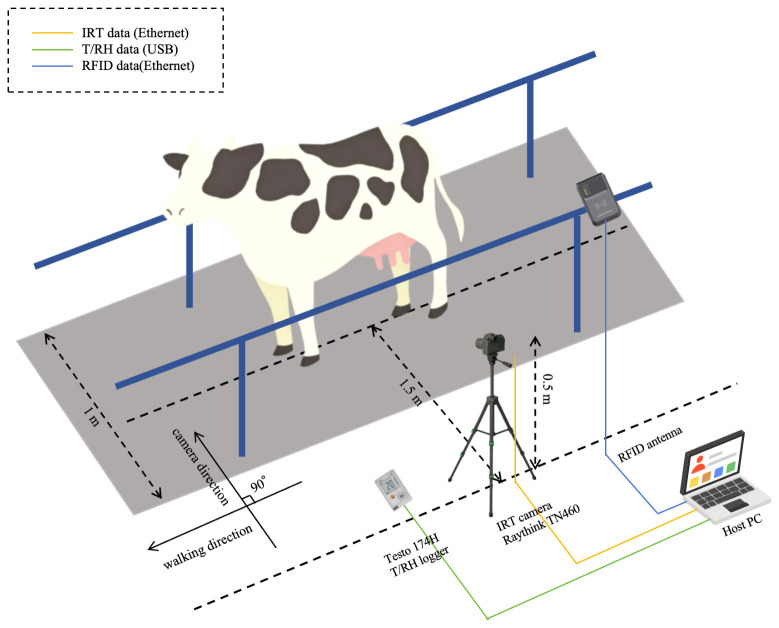
Schematic of the on-farm IRT data acquisition setup. Cows walked at their natural pace (0.3–0.5 m/s) through a dedicated 1 m-wide cattle passage. A Raythink TN460 thermal camera was mounted on a tripod at 0.5 m height and 1.5 m from the passage midline, with its optical axis kept perpendicular to the cow body. An RFID antenna at the passage entrance performed automatic cow identification; a Testo 174H logger recorded ambient temperature and relative humidity in real time. Operators stood at >3 m from the passage to avoid stress-induced thermoregulatory fluctuations. All streams were synchronized through a single host PC.

To analyze the full-body thermal characteristics of dairy cows, a high-resolution infrared thermography system was established. The primary sensor was a Raythink-TN460 (Yantai, Shandong, China) thermal camera (spectral range: 7.5–14 μm; NETD⩽40 mK). The camera streams 14-bit radiometric video in the manufacturer’s native .irv format, from which per-frame absolute-temperature radiometric .csv matrices are later exported via the manufacturer-supplied temperature analysis software TI Studio (V1.0.1). The camera was mounted on a tripod adjacent to a dedicated cattle passage to minimize motion blur and was powered by a portable supply to maintain operational stability. To synchronize environmental and identification data, two additional systems were integrated into the acquisition pipeline: a Testo 174H (Shanghai, P.R. China) recorder (*T*: −20–70 °C; RH: 0–100%) was positioned near the passage to log ambient conditions in real time. An RFID sensor system was deployed at the passage entrance to enable automatic cow identification. All devices were interfaced with a central host computer to ensure synchronous data acquisition across the thermal, environmental, and identification streams as shown in Figure 3.

The camera was used under its factory-calibrated radiometric mode; no in-field blackbody recalibration was performed during the acquisition campaign. The reflected (apparent ambient) temperature parameter of the radiometric correction model was set equal to the concurrent ambient temperature logged by the Testo 174H, an assumption justified by the diffuse barn-interior imaging geometry at incidence <10° and the exclusion of direct solar radiation, artificial ventilation, and high-intensity illumination from the acquisition window. Surface emissivity was set to ε=0.98, the accepted value for short-haired pigmented bovine skin; per-cow emissivity was not measured. The object-to-sensor distance was geometrically fixed at 1.5 m and input to the SDK atmospheric correction together with $T_{\mathrm{amb}}$ and RH from the Testo 174H. Single-pixel noise robustness of the temperature extraction is addressed by per-pixel clipping at 42 °C and a minimum mask-area gate $K_{\min}=200$ px. The absence of in-field blackbody recalibration and of per-cow emissivity measurement limits the absolute accuracy of the reported temperatures to the factory-spec NETD (≤40 mK) plus the residual emissivity mismatch (within ±0.1 °C across the bovine skin emissivity range 0.96–0.98); the relative quantity $\Delta T=T_{\mathrm{udder}}-T_{\mathrm{eye}}$ on which the diagnosis rests is by construction insensitive to common-mode calibration offset.

#### 2.1.2. Data Acquisition Protocol

Data collection was conducted at the QingHe Grass Dairy Cattle Farm in Hohhot, Inner Mongolia, China (40.167080° N, 111.770479° E) during May 2025. To minimize circadian thermal variations, recording sessions were restricted to 9:00–11:00 a.m. under standardized conditions (*T*: 15–25 °C; RH: 50–70%). Potential sources of thermal interference—including artificial ventilation, direct solar radiation, and high-intensity illumination—were strictly controlled.

The acquisition window was selected with reference to the farm’s fixed twice-daily milking schedule. All IRT recordings were performed during the 09:00–11:00 window, which corresponds to approximately 3 h after the morning milking and approximately 5 h before the next (evening) milking. All acquisitions therefore fell into the inter-milking interval rather than immediately before or immediately after milking, in order to (i) avoid the short post-milking cooling phase during which residual milk and milking-procedure manipulation introduce transient temperature variation, and (ii) avoid the immediately pre-milking interval during which severe udder engorgement may transiently elevate the baseline udder temperature. The milking-relative timing was therefore approximately uniform across all 240 enrolled cows.

No active cleaning of the udder was performed prior to imaging, in order to preserve the non-invasive nature of the protocol; however, to guarantee valid image quality, the image-quality filtering discarded all frames where the udder surface was visibly soiled or wet. This exclusion was applied uniformly across both SCC^+^ and SCC^−^ cows. For the 40-cow blind diagnostic cohort, cows whose udders were visibly soiled or moist at the time of passage were re-imaged on a subsequent passage during the same morning session, so that the diagnostic test frames came exclusively from cows with visibly dry and uncontaminated udder surfaces.

No enforced pre-imaging acclimation period was applied: the acquisition was integrated into the cows’ normal daily movement between the lying and feeding areas, with the natural walking pace and the deliberately maintained operator distance acting as the de-facto stress-avoidance strategy. Because the recording window (09:00–11:00) lay several hours after the morning milking, transient milking-procedure-induced peripheral vasodilation of the udder is expected to have subsided. The animals had been housed indoors for the entire preceding period, so abrupt indoor–outdoor ambient transitions immediately before imaging are not expected.

The thermal camera was configured with an emissivity (ϵ) of 0.98, an infrared resolution of 640×512 pixels, and a frame rate of 25 fps. The acquisition mode was specifically calibrated for animal physiological temperatures.

#### 2.1.3. Experimental Procedure and Ground Truth Acquisition

A diverse cohort of 240 Holstein cows, varying in age, parity, and lactation stage, was utilized for the study. The camera was positioned approximately 1.5 m from the passage midline, with its optical axis kept nearly perpendicular to the cow’s body surface to minimize directional-emissivity effects and distance-induced measurement bias. Cows walked individually through the passage at their natural pace (typically 0.3–0.5 m/s), without any physical restraint or external pacing applied. Operators maintained a distance from the animals throughout the recording to avoid stress-induced thermoregulatory fluctuations, ensuring that the captured thermal signatures reflected each cow’s resting physiological state.

To establish the diagnostic ground truth, composite milk samples collected from each cow were subjected to SCC analysis. Cows with a composite SCC $>2\times 10^{5}$ cells/mL were categorized as positive for subclinical mastitis, in accordance with International Dairy Federation (IDF) recommendations and previous studies [32]. It should be noted that composite-milk SCC is a widely used screening indicator rather than a definitive (bacteriological or quarter-level) reference. Because SCC can be modulated by parity, lactation stage and other physiological factors, and because a focal single-quarter infection may be diluted within a composite sample, this cow-level SCC labelling may introduce a degree of misclassification; its consequences are discussed in Section 5. These SCC results, alongside metadata such as health status and recording timestamps, were logged concurrently with the thermal video streams to ensure precise synchronization between thermal signatures and physiological health status.

We note that, by construction, all 20 mastitis-positive cows in the diagnostic cohort were classified as subclinical mastitis (SCM) under this criterion: none presented clinical signs (visible udder inflammation, abnormal milk appearance, or systemic fever) at the time of imaging. The present study therefore addresses the screening of subclinical mastitis specifically; the discrimination of clinical mastitis (CM) from SCM is beyond the scope of the current cohort and is identified as future work (Section 5).

The passage was bounded by side rails that constrained the cow’s lateral position to within ±20 cm of the passage midline (Figure 3); no physical restraint device (stanchion, head-gate, or chute) was applied, in keeping with the non-invasive nature of the protocol. Spontaneous behaviors such as head rotation, pausing, occasional leaning against the rails, and hind-leg occlusion of the udder were not constrained during acquisition; instead, they were addressed downstream in the pipeline. Head rotation, which is the dominant posture-induced confound on ΔT analysis (Section 1), is explicitly classified out by the DAT-YOLO26-pose gateway (Section 3.3.2), whose quantitative contribution is reported in Section 3.5. Hind-leg occlusion of the udder is handled by (i) discarding frames in which the udder instance-segmentation mask has area below $K_{\min}=200$ px (Section 2.4.4), and (ii) extracting the maximum per-cow temperature among all valid frames, eliminating bias caused by any single occluded frame. Pauses and brief stops are tolerated by the per-frame nature of the extraction pipeline and do not require any additional handling step.

#### 2.1.4. Ethics Statement

This study was conducted in accordance with the guidelines of the Laboratory Animal Welfare and Ethics Committee of Inner Mongolia Agricultural University (Approval No. NND2025154).

### 2.2. Data Preparation and Annotation

A total of 240 Holstein cows were enrolled in this study. To evaluate the end-to-end mastitis detection performance of the proposed pipeline, 40 cows (20 healthy and 20 diagnosed with mastitis based on SCC testing) were randomly selected and reserved as an independent diagnostic test cohort, ensuring that the final diagnostic evaluation was conducted on subjects entirely unseen during model training and validation. The IRT video streams collected from the remaining 200 cows were preprocessed and filtered to construct a refined dataset consisting of over 7000 high-quality pseudo-color .jpg renderings. To support the multi-task learning objectives of this study, two distinct annotation protocols were implemented using the X-AnyLabeling 3.0 labeling tool. To evaluate the generalization performance and stability of the DAT-YOLO26 model, the data-partitioning protocol used in this study operates at two distinct levels of isolation, which we now state explicitly so that the reader can distinguish which evaluation metrics are immune to inter-frame leakage and which are not. The outer split was performed at the cow level: the 40-cow blind diagnostic cohort (20 SCC^+^ and 20 SCC^−^ cows) was reserved a priori and contains no animal present in any training, validation, or inner-test image. The inner 7:2:1 split of the remaining 200 cows, in contrast, was performed at the frame level: the >7000 annotated images were pooled across the 200 cows and randomly partitioned into training, validation, and inner-test subsets. We acknowledge that the high inter-frame correlation of IRT video streams may cause the keypoint and segmentation metrics computed on the inner-test subset (Section 3.2, Section 3.3 and Section 3.4) to be slightly optimistic; these metrics are used in this study only for architecture comparison and hyper-parameter tuning, not as the basis of any diagnostic claim. All diagnostic figures and the confusion matrix reported in Section 3.5 were obtained exclusively from the 40-cow blind cohort, which is cow-level disjoint from any data used during model development and is therefore not subject to inter-frame leakage. Retraining the model under a fully cow-level inner-split protocol, so as to provide leakage-free intermediate metrics as well, is identified as near-term follow-up work (Section 5). The cow and image counts of each subset are summarized in Table 1.

#### 2.2.1. Pose Estimation Annotation

**Figure 4:**
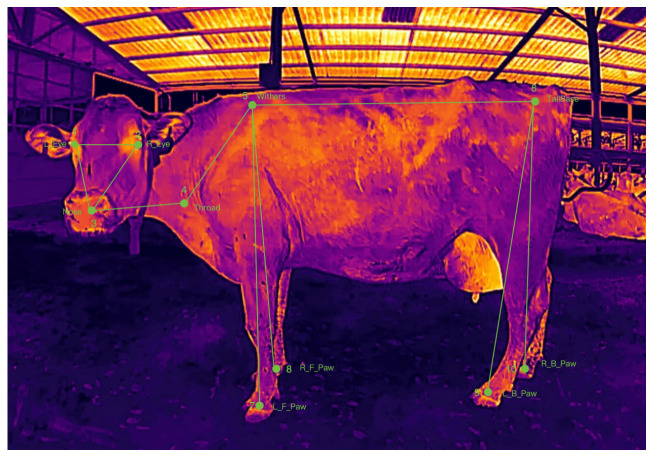
Illustration of a cow’s IRT image with detailed anatomical keypoint annotations.

For the bovine pose recognition task, we defined a skeletal topological map consisting of key anatomical landmarks. These keypoints facilitate the identification of the cow’s spatial orientation and the validation of the posture required for accurate thermal analysis. In the keypoint annotation scheme, we only focus on the spatial relationship between the cow’s head and torso. Therefore, the number of annotated keypoints is intentionally simplified. Unlike conventional schemes that label all limb joints (e.g., elbows and knees) [33], only a subset of core anatomical keypoints is selected and annotated, comprising bilateral eye canthi (L_Eye, R_Eye), nose, throat, withers, tailbase, and four hoof positions (L_F_Paw, R_F_Paw, L_B_Paw, R_B_Paw). This strategy significantly reduces manual annotation workload and improves model training efficiency for posture estimation tasks. The definitions and indices of these keypoints are summarized in Table 2. Figure 4 illustrates a representative IRT image with annotated anatomical keypoints.

In addition to keypoint localization, each image frame was simultaneously assigned a posture category label through manual inspection. Two mutually exclusive posture classes were defined: (1) lateral forward-moving posture, referring to frames in which the cow moves straight ahead with the head and longitudinal body axis aligned perpendicular to the camera’s optical axis, and (2) head-turning posture, referring to frames in which the cow’s head is rotated horizontally toward or away from the camera, resulting in a deviation from the lateral forward-moving posture. These binary class labels constitute the ground-truth dataset for training and evaluating the posture classification algorithms described in Section 3.3.

#### 2.2.2. Instance Segmentation Annotation

To enable precise pixel-level temperature extraction, instance segmentation masks were generated for the primary regions of interest (ROIs). Annotators manually delineated the boundaries of the ocular and mammary regions to separate the biological targets from the complex farm background. The class labels and their corresponding descriptions for the segmentation task are summarized in Table 3.

### 2.3. Dairy Cow Pose Estimation and Instance Segmentation Framework

#### 2.3.1. YOLO26 Architecture Overview

YOLO26 [29,30] is adopted as the baseline framework upon which the proposed DAT-YOLO26 variants are built. As the latest iteration of the YOLO family, it incorporates several architectural and training refinements that align with the specific requirements of in-barn IRT analysis—small low-contrast targets, accurate instance masks for temperature extraction, and reliable keypoint localization for posture filtering. Only the features directly relevant to the present study are summarized below; readers are referred to [29,30] for the complete description.

Following the end-to-end design first introduced in YOLOv10 [34], YOLO26 generates final predictions without Non-Maximum Suppression (NMS), and additionally removes the Distribution Focal Loss (DFL) module. This shortens the inference pipeline, simplifies model export to edge runtimes, and improves robustness when multiple animals are present in the field of view in less constrained acquisition settings. YOLO26 integrates ProgLoss and STAL (Stable Advanced Loss) to improve sensitivity on small or weakly contrasted objects. This property is directly relevant to the present task: in lateral IRT views, the ocular canthus appears as a small region with limited thermal contrast against the surrounding facial skin, while the udder boundary suffers from “thermal blur” caused by lateral heat conduction.

For segmentation, YOLO26 adopts a semantic segmentation auxiliary loss together with an upgraded multi-scale proto module, yielding tighter mask boundaries—a prerequisite for “pure” temperature extraction at the edges of the eye and udder regions (Section 2.4.4). For pose estimation, Residual Log-Likelihood Estimation (RLE) is incorporated to improve keypoint localization under labeling uncertainty, which directly benefits the simplified ten-keypoint scheme defined in Section 2.2.1.

YOLO26 also introduces the MuSGD optimizer (a hybrid of SGD and Muon-style orthogonalized updates), which is used in our training and detailed in Section 2.5. Additional features such as oriented bounding box (OBB) detection with angle loss are not exploited in this work and are therefore omitted from this overview.

Collectively, these properties make YOLO26 a suitable baseline for the DAT-augmented variants (DAT-YOLO26-pose and DAT-YOLO26-seg) introduced in Section 2.3.2.

#### 2.3.2. Proposed Improvement: Deformable Attention Transformer (DAT) Integration

To address the specific challenges of bovine thermal imaging—namely, the presence of low-contrast thermal boundaries and complex background interference—we propose DAT-YOLO26, which integrates a Deformable Attention Transformer (DAT) into the backbone of the YOLO26 architecture.

Standard Vision Transformers (ViTs) rely on global self-attention [35], which incurs high computational costs and tends to focus on irrelevant background clutter. To mitigate these limitations, the Deformable Attention (DA) mechanism—core to the DAT model—enables dynamic adjustment of the receptive field toward the most informative regions of the cow’s anatomy. Unlike rigid window partitioning or dense global attention, DA adaptively adjusts sampling points based on image content, reducing computational overhead while enhancing focus on critical features. Its core innovation lies in a deformable attention module that learns spatial offsets to flexibly adjust the receptive field; by sharing offset parameters across queries and incorporating deformable relative position bias, DAT achieves more flexible spatial modeling than conventional window-based transformers, with improved efficiency and accuracy. Specifically, DA enhances spatial perception in two key ways for bovine thermal imaging: it captures fine-grained geometric features of the cow’s posture (essential for lateral forward-moving posture classification) and effectively separates the cow from thermal noise generated by barn infrastructure, thereby maintaining high segmentation accuracy.

**Figure 5:**
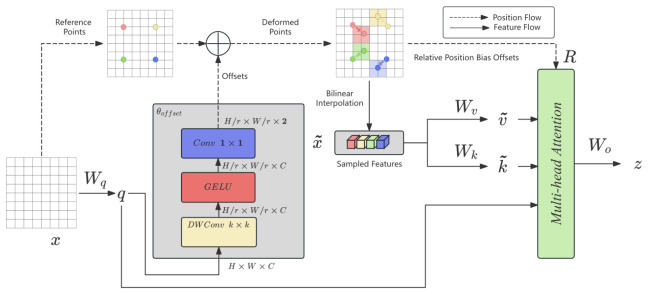
An illustration of Deformable Attention mechanism. The four colors (red, yellow, green, and blue) in the reference-point, deformed-point, and sampled-feature panels do not denote different categories; they mark four representative sampling points so that each point can be visually tracked as it is shifted by its learned offset and finally gathered into the sampled features $\tilde{x}$. Within the $\theta_{\mathrm{offset}}$ sub-network, the colored blocks (depth-wise convolution, GELU activation, and 1×1 convolution) and the multi-head attention block are shaded only to distinguish the different operations and carry no additional meaning.

The DAT mechanism further enhances standard multi-head attention by enabling content-aware spatial sampling of key/value features. Its structural pipeline is illustrated in Figure 5, and its mathematical formulation is detailed as follows:

Input Projection and Query Initialization: Given an input feature map $x\in R^{H\times W\times C}$, a linear projection with weight $W_{q}$ generates the query feature:

(2) $$q=x\cdot W_{q}\in R^{H\times W\times C}$$

The query q serves two critical purposes: (1) it initiates the offset prediction branch to learn adaptive sampling locations; (2) it directly participates in the final attention computation.

Offset Prediction Branch: To learn the spatial offsets Δp, the query is processed by a lightweight convolutional branch consisting of depthwise convolution, activation, and projection:

1. Downsampling: A depthwise convolution DWConv(k×k,stride=r) reduces the spatial resolution of q.
2. Activation: A GELU activation introduces non-linearity:
  (3) $$f_{\mathrm{act}}=\mathrm{GELU}\left(f_{\mathrm{dw}}\right)$$
3. Offset Regression: A 1×1 convolution regresses the 2D spatial offsets. To stabilize training, offsets are scaled by a factor *s*:
  (4) $$\Delta p_{\mathrm{scaled}}=s\cdot \tanh\left(\Delta p\right)\in R^{\frac{H}{r}\times \frac{W}{r}\times 2}$$

Deformed Sampling and Feature Extraction: The predicted offsets are applied to uniform reference points $p_{\mathrm{ref}}$ to generate deformed sampling points $p_{\mathrm{def}}$:

(5) $$p_{\mathrm{def}}=p_{\mathrm{ref}}+\Delta p_{\mathrm{scaled}}$$

Subsequently, bilinear interpolation ϕ is used to sample the original feature map x at these deformed locations, yielding sampled features $\tilde{x}$:

(6) $$\tilde{x}=\phi \left(x,p_{\mathrm{def}}\right)\in R^{N_{\mathrm{sample}}\times C}$$

where $N_{\mathrm{sample}}$ denotes the number of deformed sampling points.

Key/Value Projection and Attention Computation: The sampled features are linearly projected to form deformable keys and values:

(7) $$\tilde{k}=\tilde{x}\cdot W_{k},\;\tilde{v}=\tilde{x}\cdot W_{v}$$

The final multi-head attention integrates the query q, deformed keys $\tilde{k}$, values $\tilde{v}$, and a deformable relative position bias *R*:

(8) $$\mathrm{Attn}=\mathrm{Softmax}\left(\frac{q\cdot {\tilde{k}}^{⊤}}{\sqrt{d}}+R\right)\cdot \tilde{v}$$

Here, d=C/M represents the dimension of each attention head, and *M* is the number of attention heads. The deformable relative position bias *R* is computed via bilinear interpolation on a pre-learned bias table *B*, aligned with the normalized relative distances between deformed and reference points [36].

Output Projection: The attended features are finally projected by a linear layer $W_{o}$ to produce the output feature z:

(9) $$z=\mathrm{Attn}\cdot W_{o}$$

#### 2.3.3. Improved DAT-YOLO26 Architecture

**Figure 6:**
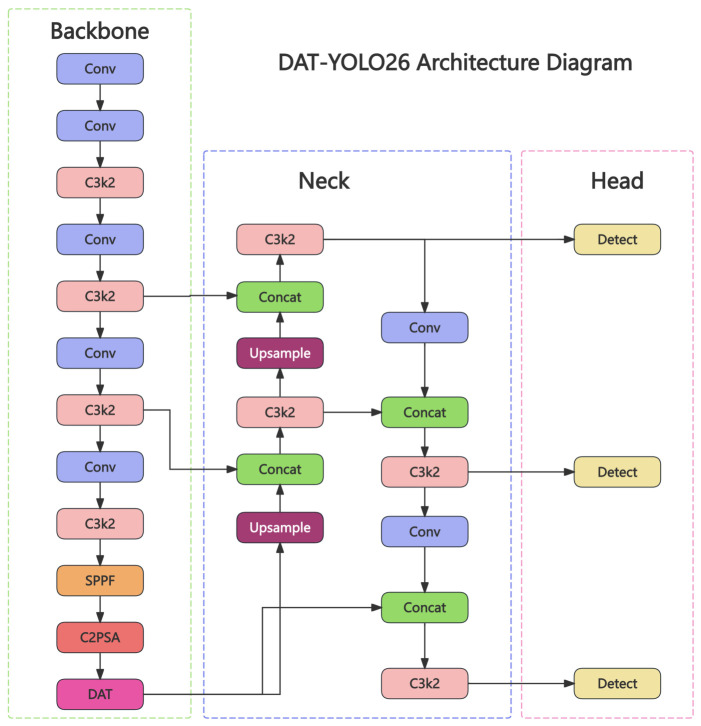
An illustration of DAT-YOLO26 architecture.

In the context of thermal imaging for dairy cows, the DAT mechanism provides two critical advantages. First, the dynamic sampling strategy allows the model to automatically concentrate its receptive field on high-information regions of the cow’s anatomy, effectively suppressing irrelevant background clutter. Second, by capturing fine-grained geometric features of the cow’s posture via adaptive spatial shifts, the DA mechanism significantly enhances spatial perception, which is crucial for accurate posture classification and segmentation tasks in complex barn environments. To address the challenges of feature extraction from infrared images in complex barn environments—such as low thermal contrast, cluttered backgrounds, and irrelevant thermal noise from barn infrastructure—we integrate a Deformable Attention Transformer (DAT) module into the backbone of the YOLO26 model, resulting in the proposed DAT-YOLO26 architecture. The original YOLO26 backbone consists of a sequence of convolutional layers, C3k2 blocks, an SPPF module, and a C2PSA module, which are responsible for extracting multi-scale semantic features from input images. However, the standard YOLO26 backbone adopts a fixed spatial sampling strategy, which is insufficient to adaptively focus on the most informative regions while suppressing background interference in infrared images. To overcome this limitation, we embed the DAT module immediately after the C2PSA layer in the YOLO26 backbone as shown in Figure 6, where the top-level semantic features (P5/32) are generated. The DAT module enables content-aware spatial sampling by learning dynamic spatial offsets, allowing the model to dynamically adjust its receptive field toward critical anatomical regions of dairy cows and effectively capture fine-grained thermal and geometric features. This integration does not alter the original multi-scale feature fusion mechanism of YOLO26, ensuring that the improved model retains the advantages of fast inference and edge deployment while enhancing feature extraction capability in complex infrared imaging scenarios. The detailed architecture of the improved YOLO26 (DAT-YOLO26) model is illustrated in Figure 6.

### 2.4. Evaluation Metrics

For comprehensive quantitative assessment of the proposed method, keypoint detection and instance segmentation tasks are evaluated using task-specific metrics. Meanwhile, common diagnostic indicators are adopted for bovine mastitis classification.

#### 2.4.1. Keypoint Detection Evaluation Metrics

The evaluation of keypoint detection draws inspiration from the metrics used in object detection, where Intersection over Union (IoU) quantifies the overlap between predicted and ground-truth bounding boxes. For keypoint detection, the core evaluation metric is Object Keypoint Similarity (OKS), which measures the similarity between predicted and ground-truth keypoints by incorporating Euclidean distance, object scale, keypoint visibility, and per-keypoint labeling uncertainty. The OKS is computed as Equation (10):

(10) $$\mathrm{OKS}=\frac{\sum_{i}\left[\exp\left(-\frac{d_{i}^{2}}{2s_{p}^{2}\sigma_{i}^{2}}\right)\cdot \delta \left(v_{i}>0\right)\right]}{\sum_{i}\delta \left(v_{i}>0\right)}$$

where $d_{i}$ is the Euclidean distance between the predicted and ground-truth coordinates of the *i*-th keypoint; $s_{p}$ denotes the object scale, defined as the square root of the bounding-box area, which normalizes the distance term for scale variations across instances of differing apparent sizes; $\sigma_{i}$ is a per-keypoint constant reflecting the inherent labeling uncertainty of the *i*-th keypoint type; and $v_{i}$ indicates the visibility status of the *i*-th keypoint, with $\delta (v_{i}>0)$ masking out non-visible keypoints from the calculation [37,38].

Based on the OKS, Average Precision (AP) is computed to evaluate keypoint detection performance. A predefined threshold *T* is used to determine whether a predicted keypoint is considered a true positive: a prediction is deemed correct if its OKS exceeds *T*. The AP metric aggregates these binary decisions across all instances and keypoints as shown in Equation (11):

(11) $$\mathrm{AP}=\frac{\sum_{m}\sum_{p}\mathrm{OKS}}{\sum_{m}\sum_{p}1},\;\mathrm{where}\;\mathrm{OKS}=\left\{\begin{array}{cc} \mathrm{OKS} & \left(\mathrm{OKS}>T\right)\\ 0 & \left(\mathrm{OKS}\leq T\right) \end{array}\right.$$

Commonly used thresholds include $mAP_{50}$ (threshold T=0.5) and $mAP_{50:95}$, which averages AP values over OKS thresholds ranging from 0.50 to 0.95 in increments of 0.05. To obtain a unified performance metric across all keypoint categories, mean Average Precision (mAP) is calculated:

(12) $$\mathrm{mAP}=\frac{\sum_{i=1}^{N}{\mathrm{AP}}_{i}}{N}$$

where *N* is the total number of keypoint categories.

#### 2.4.2. Instance Segmentation Evaluation Metrics

The performance of the DAT-YOLO26-seg instance segmentation module is evaluated using the standard COCO (Common Objects in Context) metrics [39]. The fundamental measure for segmentation accuracy is the Mask Intersection over Union (IoU), which quantifies the spatial overlap between the predicted mask ($M_{p}$) and the ground-truth mask ($M_{gt}$):

(13) $${\mathrm{IoU}}_{mask}=\frac{|M_{p}\cap M_{gt}|}{|M_{p}\cup M_{gt}|}$$

Based on the IoU, the following primary metrics are employed to assess the reliability of ocular and udder region extraction.

The Mean Average Precision (mAP) is the primary metric used to evaluate the trade-off between precision and recall across multiple Intersection over Union (IoU) thresholds. The calculation follows a hierarchical process:

For a given IoU threshold *k*, detections are classified as True Positives (TP) if IoU≥k, and False Positives (FP) otherwise. Precision (*P*) and Recall (*R*) are defined as:

(14) $$P\left(k\right)=\frac{TP(k)}{TP(k)+FP(k)}\;\;\;,\;R\left(k\right)=\frac{TP(k)}{TP(k)+FN(k)}$$

where FN denotes False Negatives (missed ground-truth objects).

The Average Precision (AP) is defined as the area under the Precision–Recall curve. To mitigate the impact of wiggles in the curve, COCO utilizes an N-point interpolation (typically 101 points) to smooth the precision:

(15) $$P_{interp}\left(r\right)=\max_{\tilde{r}\geq r}P\left(\tilde{r}\right)$$

The AP for a specific category is then calculated using the integral of the interpolated precision over the recall range [0,1]:

(16) $$AP=\int_{0}^{1}P_{interp}\left(r\right)\;dr\approx \frac{1}{N}\sum_{i=1}^{N}P_{interp}\left(r_{i}\right)$$

The Mean Average Precision (mAP) is the arithmetic mean of the AP calculated across all *C* categories (in this study, ocular and udder regions):

(17) $$mAP=\frac{1}{C}\sum_{j=1}^{C}AP_{j}$$

To evaluate the model’s localization robustness, the final metric is averaged over ten IoU thresholds from 0.5 to 0.95 with a step size of 0.05:

(18) $$mAP_{50:95}=\frac{1}{10}\sum_{k\in \{0.5,0.55,\ldots ,0.95\}}mAP\left(k\right)$$

Specifically, $mAP_{75}$ refers to the mean average precision calculated at the stringent threshold of k=0.75, serving as a benchmark for high-precision anatomical segmentation.

#### 2.4.3. Diagnostic Classification Metrics

To assess the performance of the proposed mastitis detection framework as a binary classification task (i.e., mastitis-positive vs. healthy), five widely adopted diagnostic metrics are used: Accuracy, Precision, Recall (Sensitivity), Specificity, and F1-score. All metrics are derived from the confusion matrix, where TP (True Positive) represents mastitis-positive samples correctly identified, TN (True Negative) denotes healthy samples correctly classified, FP (False Positive) indicates healthy samples misdiagnosed as mastitis-positive, and FN (False Negative) refers to mastitis-positive samples missed by the model.

Accuracy measures the overall proportion of correctly classified samples among all test instances, reflecting the global consistency between model predictions and true labels:

(19) $$Accuracy=\frac{TP+TN}{TP+TN+FP+FN}$$

While accuracy provides a straightforward summary of performance, it can be misleading in imbalanced datasets, where one class (e.g., healthy cows) significantly outnumbers the other.

Precision quantifies the proportion of predicted positive cases that are truly positive, evaluating the reliability of the model’s positive diagnoses:

(20) $$Precision=\frac{TP}{TP+FP}$$

In practical breeding applications, high precision is critical to avoid unnecessary interventions or treatments triggered by false alarms.

Recall, also referred to as sensitivity in clinical diagnostics, measures the proportion of actual positive cases that are correctly detected by the model:

(21) $$Recall\left(Sensitivity\right)=\frac{TP}{TP+FN}$$

For early mastitis screening, high sensitivity is essential, as it minimizes the risk of missing affected cows, thereby enabling timely intervention to reduce economic losses and animal welfare risks.

Specificity evaluates the model’s ability to correctly identify negative (healthy) samples, quantifying the proportion of true negatives among all predicted negative cases:

(22) $$Specificity=\frac{TN}{TN+FP}$$

High specificity ensures that healthy cows are not incorrectly flagged as mastitis-positive, which is vital for reducing farmer workload and unnecessary stress on animals.

The F1-score is the harmonic mean of precision and recall, providing a balanced measure that considers both false positives and false negatives:

(23) $$F1-score=2\times \frac{Precision\times Recall}{Precision+Recall}$$

This is particularly useful when the dataset is imbalanced, offering a more robust assessment of overall classification performance than accuracy alone.

Together, these metrics provide a comprehensive evaluation of the proposed framework, capturing different aspects of its performance in the context of bovine mastitis screening, from overall accuracy to clinical reliability and practical utility.

#### 2.4.4. Temperature Acquisition and Diagnostic Criteria for Cow Mastitis

Based on physiological characteristics and the relevant literature, the temperature difference between the udder and the eye serves as a critical indicator for identifying subclinical mastitis in dairy cows. Following Sathiyabarathi et al. [10], a threshold of ΔT > 0.8 °C is adopted as the criterion for mastitis diagnosis.

To establish a quantitative diagnostic criterion, a binary classification function $S_{\mathrm{mastitis}}$ is defined as Equation (24):

(24) $$S_{\mathrm{mastitis}}=\left\{\begin{array}{cc} 0, & T_{\mathrm{udder}}-T_{\mathrm{eye}}\leq 0.8\\ 1, & T_{\mathrm{udder}}-T_{\mathrm{eye}}>0.8 \end{array}\right.$$

where $S_{\mathrm{mastitis}}$ represents the diagnostic result; a value of 0 indicates a normal health status, while a value of 1 indicates mastitis-positive. $T_{\mathrm{eye}}$ denotes the maximum ocular surface temperature, and $T_{\mathrm{udder}}$ denotes the maximum udder skin surface temperature.

To ensure the accuracy of temperature measurement, the maximum temperature values are extracted from the thermal infrared images of the eye and udder regions. The $T_{\mathrm{eye}}$, which reflects the core body temperature of the cow, is calculated by taking the maximum value across all valid eye images:

(25) $$T_{\mathrm{eye}}={\mathrm{Max}}_{n\in [1,N_{e}]}\left({\mathrm{Max}}_{i,j\in [1,S_{e}]}\left(T(x_{i},y_{j})\right)\right)$$

Similarly, the $T_{\mathrm{udder}}$ is extracted by computing the maximum value within the udder region across all valid thermal images:

(26) $$T_{\mathrm{udder}}={\mathrm{Max}}_{m\in [1,N_{u}]}\left({\mathrm{Max}}_{i,j\in [1,S_{u}]}\left(T(x_{i},y_{j})\right)\right)$$

##### Variable Definition

- $T(x_{i},y_{j})$: The temperature value at coordinate $\left(x_{i},y_{j}\right)$ in the thermal infrared matrix.
- $N_{e}$: The total number of thermal infrared images containing the eye region.
- $N_{u}$: The total number of thermal infrared images containing the udder region.
- $S_{e}$: The number of elements (pixels) in the eye temperature matrix of the thermal image.
- $S_{u}$: The number of elements (pixels) in the udder temperature matrix of the thermal image.

The use of the maximum operator is consistent with prior IRT-based mastitis detection studies [10,11,12,13,14,15,16], where the highest temperature within a segmented region is taken to represent its physiologically informative hot zone (the well-vascularized ocular canthus and the most inflamed udder quarter). To mitigate the inherent sensitivity of the maximum operator to single-pixel outliers, the following safeguards were applied in our pipeline: (i) DAT-YOLO26-seg produces masks that tightly encapsulate the target tissue and exclude reflective background pixels; (ii) frames in which the eye or udder mask contains fewer than $K_{\min}$ pixels (set to 200 in this study) are discarded as low-confidence detections; and (iii) per-pixel temperatures exceeding 42 °C—a physiologically implausible threshold for bovine surface tissue—are clipped prior to the maximum operation to suppress specular-reflection artifacts. A sensitivity analysis on the validation set further confirmed that replacing the maximum with the per-frame 95th-percentile altered $T_{\mathrm{eye}}$ and $T_{\mathrm{udder}}$ by less than 0.15 °C on average, indicating that the diagnostic conclusions are largely insensitive to the choice of pooling statistic.

This diagnostic framework establishes a clear threshold for mastitis detection, leveraging the physiological correlation between eye and udder temperatures to provide a consistent, non-invasive assessment strategy for dairy cattle health management. The complete per-cow diagnostic procedure—from raw thermal video through the posture and mask-area gateways to the final differential-temperature decision—is summarized in Algorithm 1.

### 2.5. Experimental Setup and Hyperparameter Configuration

Table 4 details the hardware and software configuration utilized for model training and testing. All experiments were conducted on the high-performance computing platform specified in this table.

The training was conducted for 100 epochs with a batch size of 64. Following the official YOLO26 training recipe [30], the initial learning rate was set to 0.00038 with a final learning rate factor of 0.882, momentum of 0.948, and weight decay of 0.00027. The MuSGD optimizer—a hybrid scheme combining SGD updates with Muon-style orthogonalized updates on convolutional weights—was employed to stabilize gradient flow during the learning of complex spatial relationships in skeletal keypoint detection. The input image resolution was set to 640 × 640 pixels, consistent with the YOLO26 pretraining configuration.
**Algorithm 1** Per-cow eye–udder differential temperature pipeline.**Require:** Raw IRT video $V={\left\{F_{t}\right\}}_{t=1}^{T}$ for one cow; ambient $T_{\mathrm{amb}}$, RH from Testo 174H;sensor–object distance d=1.5 m; emissivity ε=0.98**Ensure:** Per-cow differential temperature $\Delta T_{\mathrm{cow}}$ and binary diagnosis1:$D_{eye},D_{udder}\leftarrow ⌀$▹ valid per-frame max $T_{eye},T_{udder}$ buffer2:**for** each frame $F_{t}\in V$ **do**3:    $T_{t}\left(x,y\right)$ ← SDKExport(*F_t_*, *T*_amb_, *RH*, *d*, *ε*)▹ 14-bit →640×512 absolute temperature matrix in °C4:    $T_{t}\left(x,y\right)\leftarrow \min\left\{T_{t}\left(x,y\right),\;42\;{}^{\circ}C\right\}$▹ clip physiologically implausible pixels5:    $K_{t}^{\mathrm{pose}}$ ← DAT-YOLO26-pose(*F_t_*)▹ 10 anatomical keypoints $K_{1}$–$K_{10}$6:    $f_{t}$ ← NormalizeKeypoints($K_{t}^{\mathrm{pose}}$)▹ 20-D bbox-normalized feature vector (Section 3.3.2)7:    $c_{t}^{\mathrm{pose}}\leftarrow {\mathrm{SVM}}_{\mathrm{RBF}}\left(f_{t}\right)$▹ posture class ∈ {lateral, head-turning}8:    **if** $c_{t}^{\mathrm{pose}}=“\mathrm{head}-\mathrm{turning}”$ **then**9:          **continue**▹ posture gateway rejects frame10:    **end if**11:    $\left(M_{t}^{\mathrm{eye}},M_{t}^{\mathrm{udder}}\right)$ ← DAT-YOLO26-seg(*F_t_*)
▹ binary masks $\in {\left\{0,1\right\}}^{640\times 512}$12:    **if** $|M_{t}^{\mathrm{udder}}|<K_{\min}=200$ **or** $|M_{t}^{\mathrm{eye}}|<K_{\min}=200$ **then**13:          **continue**▹ mask-area gateway rejects frame14:    **end if**15:    $T_{t}^{\mathrm{eye}}\leftarrow \mathrm{Max}\left(\{T_{t}\left(x,y\right):M_{t}^{\mathrm{eye}}\left(x,y\right)=1\}\right)$16:    $T_{t}^{\mathrm{udder}}\leftarrow \mathrm{Max}\left(\{T_{t}\left(x,y\right):M_{t}^{\mathrm{udder}}\left(x,y\right)=1\}\right)$17:    $D_{eye}\leftarrow D_{eye}\cup \left\{T_{t}^{\mathrm{eye}}\right\}$18:    $D_{udder}\leftarrow D_{udder}\cup \left\{T_{t}^{\mathrm{udder}}\right\}$19:**end for**20:**if** 
$|D_{eye}|<N_{\min}=5$ 
**or** 
$|D_{udder}|<N_{\min}=5$ 
**then**21:    **return flag_reimage**▹ insufficient valid frames22:**end if**23:$\Delta T_{\mathrm{cow}}\leftarrow Max\left(D_{udder}\right)-Max\left(D_{eye}\right)$▹ multi-frame aggregation, Max across valid frames24:**return** $\Delta T_{\mathrm{cow}}$, $\left[\;\Delta T_{\mathrm{cow}}>0.8\;{}^{\circ}C\;\right]$▹ Equation (24)

## 3. Experimental Results and Analyses

In this section, we present and analyze the performance of the proposed DAT-YOLO26 framework, including both the pose estimation and instance segmentation tasks, as well as the final diagnostic performance for bovine mastitis detection. All experiments are conducted on the collected dairy cow thermal infrared dataset, with performance evaluated using the metrics defined in Section 2.4.

### 3.1. Baseline Model Selection

In this work, YOLO26m is selected as the baseline network for pose estimation and instance segmentation. Compared with lightweight small models (YOLO26n) and large deep models (YOLO26l/x), YOLO26m achieves a balanced trade-off between feature representation capability, computational consumption and on-site deployment practicability. It can effectively capture low-contrast thermal features, weak boundary information and fine anatomical keypoints in dairy cow infrared images, while avoiding excessive parameters and high inference latency. On the basis of YOLO26m, we embed the Deformable Attention Transformer (DAT) to construct DAT-YOLO26-pose and DAT-YOLO26-seg for cow posture recognition and key region instance segmentation, respectively.

To fully verify the effectiveness of the proposed method, extensive comparison experiments are conducted with mainstream YOLO series models, including YOLOv8, YOLOv9, YOLOv10, YOLO11, YOLO12 and original YOLO26m. Quantitative evaluations are comprehensively performed in terms of keypoint detection accuracy, instance segmentation quality, model parameter scale and computational complexity (GFLOPs).

### 3.2. Comparison of Pose Estimation Performance Across Different YOLO Series Models

The pose estimation task focuses on high-precision keypoint localization and posture feature extraction. To comprehensively evaluate performance, $mAP_{50}$, $mAP_{75}$, and $mAP_{50:95}$ were adopted as the primary evaluation metrics. Additionally, model parameters (Params) and GFLOPs were introduced to assess computational efficiency and deployment feasibility. The detailed quantitative results are summarized in Table 5.

As illustrated in Table 5, the DAT-YOLO26m-pose model achieves superior performance in bovine pose estimation, consistently outperforming both the baseline YOLO26m and standard YOLO series models. Notably, the integration of the Deformable Attention Transformer (DAT) module contributed to an absolute improvement of 1.8 percentage points in $mAP_{50:95}$ (from 0.796 to 0.814) and 2.8 percentage points in $mAP_{75}$ (from 0.824 to 0.852).

These ablation results confirm that the DAT module significantly enhances the model’s capacity for fine-grained keypoint localization, particularly under the low-contrast conditions typical of infrared imaging.

While the DAT-enhanced architecture introduces a negligible increase in computational cost (+0.3 M Params and +0.4 GFLOPs), its total operation count (73.5 GFLOPs) remains substantially lower than that of YOLOv9m and YOLOv10m. This favorable balance between accuracy and computational cost provides a foundation for future deployment on resource-constrained edge devices; comprehensive on-device benchmarking is left to subsequent work.

### 3.3. Posture Classification Algorithm Based on Keypoint Detection

After obtaining precise anatomical landmarks via the DAT-YOLO26-pose model, we implemented a posture validation stage to ensure only frames classified as lateral forward-moving posture are utilized for thermal analysis. This stage is critical because off-axis viewing angles and head deflections introduce emissivity variations and measurement errors due to changes in atmospheric path length in IRT. We compared a geometrically driven Threshold-Based Method with a machine-learning-driven SVM-based method to identify the most robust “gatekeeper” for the diagnostic pipeline.

#### 3.3.1. Threshold-Based Geometric Validation via Perspective and Symmetry Constraints

The threshold-based method utilizes a deterministic gating logic based on the spatial arrangement and axial alignment of the bovine skeleton ($K_{1}$–$K_{10}$). In our experimental setup, where the camera is positioned perpendicular to the transit passage, an ideal posture is defined as the cow moving straight ahead without head deflection or torso twisting. Three primary geometric features were extracted:

1. Mid-Sagittal Axial Collinearity ($\theta_{axial}$): When a cow moves straight without turning its head, the nose ($K_{3}$), throat ($K_{4}$), and withers ($K_{5}$) should align linearly in the 2D projected image plane. This indicates that the mid-sagittal plane of the head is parallel to the camera sensor. The collinearity is assessed by the interior angle formed by the vectors connecting $K_{3}$, $K_{4}$, and $K_{5}$:
  - Criterion: $\theta_{axial}\in \left[175{}^{\circ},180{}^{\circ}\right]$.
  - Significance: This ensures the head is not deflected laterally, which is essential for accurate thermal quantification of the ocular canthus ($K_{1},K_{2}$).
2. Perspective Foreshortening of the Cranial Segment ($D_{kt}$): The projected Euclidean distance between the nose ($K_{3}$) and the throat ($K_{4}$), denoted as $D_{kt}$, serves as a high-sensitivity indicator of head rotation. According to the principles of perspective projection, $D_{kt}$ reaches its maximum when the head is parallel to the image plane.
  - Criterion: $D_{kt}\geq \rho$ (where ρ is a normalized distance threshold. ρ was empirically determined as 0.86 based on the 10th percentile of $D_{kt}$ values observed in validated lateral-forward posture frames from the training set. Sensitivity analysis confirmed that varying ρ within 0.05 resulted in less than 2% change in posture classification accuracy on the validation set, indicating robustness to this parameter choice).
  - Significance: Any lateral rotation of the head causes the 3D head–neck vector to undergo perspective foreshortening in the 2D plane. Monitoring $D_{kt}$ allows the system to exclude frames with skewed angular emissivity.
3. Longitudinal Symmetry of Limbs ($R_{limb}$): To ensure the torso is not tilted or twisted relative to the passage centerline, the symmetry between the front and rear limb-to-axis distances was evaluated. We calculated the average projected distance from the front hooves ($K_{7},K_{8}$) to the withers ($K_{5}$), denoted as $L_{front}$, and from the back hooves ($K_{9},K_{10}$) to the tail base ($K_{6}$), denoted as $L_{back}$.
  - Criterion: $R_{limb}=L_{front}/L_{back}$, with a valid range of [0.90,1.10].
  - Significance: In a stable, non-twisting forward gait, the projected distances $L_{front}$ and $L_{back}$ remain consistent due to the constant camera-to-subject distance. A deviation in $R_{limb}$ indicates a lateral stride or body rotation, which distorts the lateral surface area of the mammary gland.

A posture is classified as a valid pose ($P_{valid}$) only if all conditions are satisfied simultaneously:

(27) $$P_{valid}=\left\{\left(\theta_{axial}\in \left[175^{\circ },180^{\circ }\right]\right)\cap \left(D_{kt}\geq \rho \right)\cap \left(R_{limb}\in \left[0.90,1.10\right]\right)\right\}$$

#### 3.3.2. SVM-Based Posture Classification

To further enhance the classification accuracy and robustness for cow posture recognition, this study trains a Support Vector Machine (SVM) [40] classifier with keypoint feature vectors, aiming to address complex nonlinear variations in cow postures.

- Feature Engineering: A 20-dimensional feature vector is constructed based on the normalized coordinates $\left(x^{\prime },y^{\prime }\right)$ of ten keypoints ($K_{1}$–$K_{10}$). All coordinate normalization operations are implemented with reference to bounding box sizes to guarantee scale invariance under varying shooting distances.
- Kernel Selection: The Radial Basis Function (RBF) kernel is adopted to project skeletal features into a high-dimensional feature space, which facilitates the distinction of subtle posture differences.
- Optimization: A 5-fold cross-validation grid search strategy is utilized to optimize hyperparameters, including the penalty factor *C* and kernel coefficient γ, so as to effectively mitigate the overfitting risk.

#### 3.3.3. Comparative Performance Analysis

Both the Threshold-Based Method and the SVM-Based Method were developed and evaluated on the posture-labeled dataset described in Section 2.2.1, in which each image frame was manually assigned one of two posture categories: lateral forward-moving posture or head-turning posture. Consistent with the overall data partitioning strategy, this labeled dataset was split into training, validation, and test subsets following a 7:2:1 ratio. Specifically, 70% of the labeled frames were used for SVM model training and threshold parameter calibration, 20% for validation and hyperparameter tuning, and the remaining 10% (comprising 700 IRT frames) served as the held-out test set for the comparative performance evaluation reported below.

The comparative performance of the two proposed posture classification algorithms is summarized in Table 6. The results demonstrate that while the Threshold-Based Method provides a baseline for pose validation, the SVM-Based Method achieves superior performance, with an accuracy of 94.29% and an F1-score of 94.19%. The Threshold-Based Method relies on fixed, deterministic inequalities (e.g., $\theta_{axial}\geq 175^{\circ }$). While physically interpretable, this approach struggles with the natural variability of cow’s movement. Subtle combinations of head tilt and body rotation can lead to “near-miss” cases where one threshold is narrowly met while another is failed.

The SVM, utilizing a Radial Basis Function (RBF) kernel, projects the 20-dimensional keypoint feature vector into a higher-dimensional space. This allows the model to learn a non-linear decision boundary that captures the complex correlations between keypoints that simple thresholds cannot account for. Dairy cows exhibit significant morphological diversity in terms of size, body condition, and gait. A fixed distance threshold for perspective foreshortening ($D_{kt}$) may misclassify a smaller cow moving correctly as “head deflected” simply due to its smaller anatomical scale. Because the SVM was trained on 70% of the posture-labeled dataset (approximately 4900 frames) and utilized normalized coordinates, it is more robust to scale variations.

The high precision (93.88%) of the SVM indicates a significantly lower rate of False Positives, ensuring that fewer “head-turning posture” or “distorted” frames are erroneously passed to the thermal analysis module. The SVM-Based Method achieved a high recall of 94.50%, whereas the Threshold-Based Method lagged at 89.00%. In a practical farm environment, a lower recall means the system would discard many potentially valid frames, leading to “gaps” in the health monitoring data. By using the SVM, the system maximizes the utility of the captured video stream, ensuring more frequent and consistent temperature samples for early mastitis detection. The significant improvement in the F1-score (a 6.08% increase over the threshold method) directly impacts the downstream diagnostic reliability. By more accurately identifying the lateral forward-moving posture, the SVM ensures that the ocular and mammary regions are captured under a consistent lateral projection geometry, thereby standardizing the imaging conditions for thermal extraction.

Considering the balance between classification accuracy and computational efficiency, the SVM-based posture classification method is finally adopted in the proposed mastitis detection pipeline. It can reliably screen out valid lateral forward-moving postures, ensuring that subsequent segmentation and temperature extraction are performed on high-quality images, thus laying a solid foundation for accurate mastitis diagnosis.

### 3.4. Comparison of Instance Segmentation Performance Across Different YOLO Series Models

The instance segmentation module (DAT-YOLO26-seg) is a critical component of the diagnostic pipeline, responsible for isolating the eye and udder regions for precise temperature extraction. To evaluate its performance, $mAP_{50}$, $mAP_{75}$, and $mAP_{50:95}$ were utilized as the primary evaluation metrics. A quantitative comparison between the proposed model and several state-of-the-art YOLO-seg variants is presented in Table 7.

As illustrated in Table 7, the DAT-YOLO26-seg model achieves superior performance in anatomical region segmentation, outperforming the standard YOLO series models across all localization thresholds. To isolate the impact of the proposed architectural enhancements, a targeted ablation analysis was conducted by comparing the DAT-enhanced version with the baseline YOLO26m-seg.

The results of this ablation demonstrate that the integration of the Deformable Attention Transformer (DAT) module yielded an absolute improvement of 3.9 percentage points in $mAP_{75}$ (from 0.843 to 0.882, a relative gain of 4.6%) and 3.6 percentage points in $mAP_{50:95}$ (a relative gain of 4.4%). In thermal infrared imaging, biological boundaries often exhibit low contrast and “thermal blurring” due to lateral heat conduction across the skin surface. The DAT module effectively addresses this challenge by dynamically adjusting its receptive field to focus on high-contrast pixels at the contours of the ocular canthus and mammary glands.

This enhanced boundary fidelity is essential for “pure” temperature extraction; it ensures that the generated masks tightly encapsulate the target physiological tissues while excluding low-temperature background noise from the barn environment. Despite the negligible increase in computational complexity (+0.3 M Params and +0.4 GFLOPs relative to the baseline), the model maintains a more efficient operation count than the YOLOv9 and YOLOv10 architectures. These findings confirm that DAT-YOLO26-seg provides the spatial precision required for consistent temperature extraction in dairy-farm settings.

The architecture-comparison metrics in Table 5 and Table 7 (Section 3.2 and Section 3.4) are reported for a single training run under the default random seed. To verify that the DAT-YOLO26 advantage is not an artifact of seed-specific initialization, we additionally repeated training over three independent runs (random seeds s∈{default,42,124}) with the frame-level inner split held identical across runs, and report the resulting mean ± standard deviation in Table 8. The cross-seed standard deviation quantifies the run-to-run noise floor of each metric, and a paired *t*-test on the three seed-matched runs (Table 8) tests whether each architectural gain exceeds that noise floor.

Across all six detection metrics, the performance improvement brought by DAT-YOLO26 shows consistent positive trends under all random seeds, with statistically significant enhancements observed across three independent experimental runs (p<0.05); the gains are largest on the stricter, high-IoU metrics (Pose mAP_75_ +0.031, Mask mAP_75_ +0.037, Mask mAP_50:95_ +0.037), indicating that the deformable attention module improves localization precision on the low-contrast ocular and mammary boundaries that dominate the high-IoU regime.

### 3.5. Overall Performance of the Mastitis Detection Pipeline

To evaluate the diagnostic performance of the proposed two-stage DAT-YOLO26 framework under practical dairy farm conditions, a blind diagnostic trial was conducted on the independent test cohort described in Section 2.2, which comprised 40 cows (20 healthy and 20 mastitis-positive, classified by composite-milk SCC at the IDF threshold, used here as an imperfect reference indicator rather than a definitive diagnosis). It should be noted that the balanced 1:1 composition was adopted to ensure statistically consistent estimates of both sensitivity and specificity. While this design does not reproduce the natural class distribution at any individual farm, the resulting 50% prevalence falls within the range reported for Chinese dairy herds—a meta-analysis of studies from 2012 to 2021 estimated the overall prevalence of subclinical mastitis at 36.4–50.2%, with the highest regional value of 72% recorded in the Inner Mongolia Autonomous Region [41]. Nevertheless, further validation on larger datasets reflecting real-world prevalence distributions is warranted to fully assess the framework’s performance in deployment scenarios.

The integrated diagnostic pipeline consists of two synergistic modules: DAT-YOLO26-pose for posture recognition and DAT-YOLO26-seg for region-of-interest segmentation. The pose estimation module serves as a critical quality gateway, filtering the input stream to retain only lateral forward-moving posture images. This pre-screening step mitigates systematic errors arising from inconsistent shooting distances and off-axis viewing angles, which typically degrade the accuracy of subsequent thermal differential calculations.

Based on the validated lateral frames, the DAT-YOLO26-seg model generates high-fidelity instance masks to extract $T_{\mathrm{eye}}$ and $T_{\mathrm{udder}}$. Following the diagnostic criteria defined in Equations (24)–(26), mastitis is diagnosed if the thermal gradient ($\Delta T=T_{\mathrm{udder}}-T_{\mathrm{eye}}$) exceeds 0.8°C. In the control condition, $T_{\mathrm{eye}}$ and $T_{\mathrm{udder}}$ were extracted from all frames in which both the eye and udder regions were successfully segmented by DAT-YOLO26-seg (i.e., mask area exceeding $K_{\min}=200$ pixels, as defined in Section 2.4.4), regardless of head orientation. This isolates the effect of posture filtering from the segmentation quality. To quantify the influence of pose constraints, we compared the diagnostic performance of the baseline YOLO26 and DAT-YOLO26 models with and without the pose screening procedure, as presented in Table 9.

As illustrated in Table 9, the integration of the posture recognition module yielded a substantial performance boost, with Accuracy and F1-score increasing by 10.00 and 10.26 percentage points, respectively. The experimental data confirm that unconstrained head movements significantly compromise diagnostic sensitivity. Specifically, when a cow rotates its head toward the thermal sensor, the reduction in sensor-to-object distance induces an artificial elevation in the apparent temperature of the ocular region.

Within our diagnostic framework, which utilizes the ocular region as a thermal baseline, such a posture-induced temperature spike narrows the recorded thermal gradient (ΔT). This phenomenon frequently leads to "false negative" outcomes, where the subtle thermal elevations characteristic of subclinical mastitis are masked by the baseline shift. By enforcing a consistent measurement geometry, the DAT-YOLO26-pose module effectively mitigates posture-associated measurement artifacts.

Consequently, the complete framework achieved 87.50% accuracy and 90.00% specificity on the 40-cow blind test cohort (95% CIs shown in Table 10). These results support the role of standardized posture acquisition as a prerequisite for non-invasive IRT-based screening in the present evaluation setting; confirmation under larger multi-farm cohorts is required before broader conclusions can be drawn (see Section 5).

#### 3.5.1. ROC Analysis and Threshold Independence

**Figure 7:**
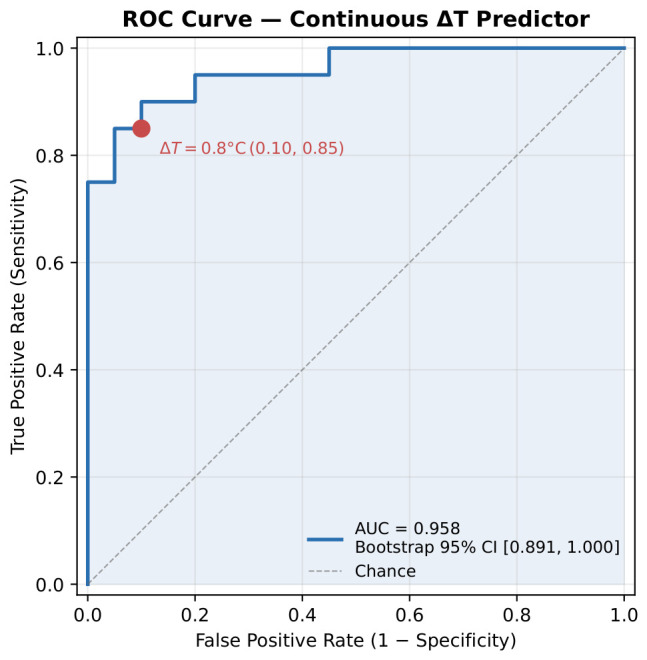
ROC curve of the proposed DAT-YOLO26 + posture-filter pipeline on the 40-cow blind diagnostic cohort, using the per-cow aggregated ΔT as a continuous score. Red dot: the deployed operating point at ΔT=0.8 °C (sensitivity 85.00%, specificity 90.00%). AUC=0.958 (Bootstrap 95% CI [0.891,1.000]).

Although the deployed decision rule is the fixed literature-derived threshold ΔT>0.8 °C, the per-cow aggregated ΔT produced by the pipeline (Section 2.4.4) can be treated as a continuous diagnostic score, enabling a full receiver-operating-characteristic (ROC) analysis. Sweeping the threshold over the empirical range of per-cow ΔT values on the 40-cow blind diagnostic cohort yields the ROC curve in Figure 7, with the deployed operating point (ΔT=0.8 °C; sensitivity 85.00%, specificity 90.00%) marked. The area under the curve is AUC=0.958 (95% bootstrap confidence interval [0.891,1.000], 2000 resamples drawn at the cow level). The empirical Youden-*J* optimum lies at ΔT=0.75 °C, close to the literature value of 0.8 °C, supporting the appropriateness of the latter for this cohort. The difference (0.05 °C) is within practical measurement uncertainty. Because AUC is threshold-independent, it complements the fixed-threshold operating-point metrics reported in Table 9 and Table 10.

#### 3.5.2. Significance Test Against the Baseline

To assess whether the DAT-YOLO26 + posture-filter pipeline produces significantly better per-cow diagnoses than the baseline configuration (baseline YOLO26 without the deformable attention module and without posture gating; cf. the performance comparison in Table 9) on the same blind cohort, we applied McNemar’s exact test to the paired binary predictions of the two pipelines over the 40 cows (Table 11). The two methods disagreed on seven cows: seven were correctly classified by the proposed pipeline but misclassified by the baseline, and none in the opposite direction. The two-sided exact McNemar test gives p=0.0156, so the improvement over the baseline is statistically significant within the present cohort.

#### 3.5.3. Class-Conditional Temperature Distributions

**Figure 8:**
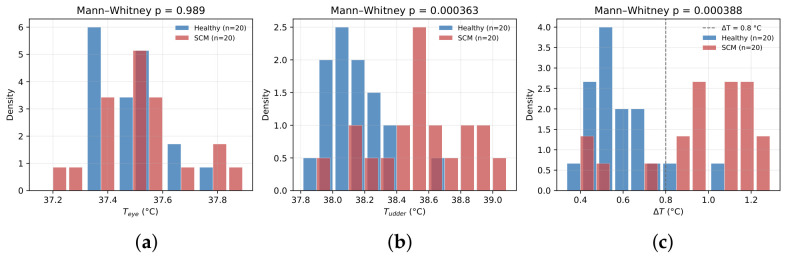
Per-cow distributions of (**a**) ocular temperature $T_{\mathrm{eye}}$, (**b**) udder temperature $T_{\mathrm{udder}}$, and (**c**) the differential $\Delta T=T_{\mathrm{udder}}-T_{\mathrm{eye}}$ for healthy (SCC^−^, n=20) and subclinical-mastitis (SCC^+^, n=20) cows on the blind diagnostic cohort. Overlaid histograms with kernel-density curves; insets show the corresponding box plots. The ΔT=0.8 °C decision threshold is marked as a vertical dashed line in (**c**). $T_{\mathrm{eye}}$ does not differ between classes (p=0.99), whereas $T_{\mathrm{udder}}$ ($p=3.6\times 10^{-4}$) and ΔT ($p=3.9\times 10^{-4}$) are both strongly discriminative (Mann–Whitney *U* test).

To expose why the eye–udder differential is used as the diagnostic variable rather than either absolute temperature alone, Figure 8 shows the per-cow distributions of $T_{\mathrm{eye}}$, $T_{\mathrm{udder}}$, and $\Delta T=T_{\mathrm{udder}}-T_{\mathrm{eye}}$ on the 40-cow blind diagnostic cohort, stratified by SCC class. The class-conditional means (± SD) and Mann–Whitney *U* tests (SCC^+^ vs. SCC^−^) are summarised in Table 12. The ocular temperature is statistically indistinguishable between the two classes (37.53±0.12 °C healthy vs. 37.53±0.17 °C SCM; U=199.0, p=0.99), confirming that $T_{\mathrm{eye}}$ functions as a stable, infection-independent thermal reference and carries essentially no diagnostic information on its own. Both the udder temperature (38.52±0.30 °C SCM vs. 38.18±0.18 °C healthy; U=68.5, $p=3.6\times 10^{-4}$) and the differential ΔT (0.98±0.27 °C SCM vs. 0.65±0.15 °C healthy; U=69.0, $p=3.9\times 10^{-4}$) discriminate the two classes strongly and to a comparable degree. The decisive advantage of ΔT is therefore not a smaller *p*-value but its mechanistic stability: subtracting the infection-independent ocular reference removes the shared animal-level and ambient baseline, so that ΔT retains its discriminative power across individuals and acquisition conditions for which the absolute $T_{\mathrm{udder}}$ would drift, whereas $T_{\mathrm{eye}}$ alone carries no signal. This pattern ($T_{\mathrm{eye}}$ uninformative; $T_{\mathrm{udder}}$ and ΔT both discriminative) provides direct empirical justification for the differential-temperature formulation adopted in this work (Section 1). The deployed threshold ΔT=0.8 °C lies between the two class means and close to the Youden-*J* optimum identified on the ROC curve (Figure 7), supporting its appropriateness for this cohort; as the reviewer notes, this optimum is cohort-specific and may shift with breed, environment, and camera settings, and the ROC analysis is reported precisely so that future deployments can re-derive the threshold for their own conditions.

Beyond the architecture × posture decomposition (Table 9), we ablated three further design choices of the temperature-extraction and posture-gating pipeline, each evaluated end-to-end on the 40-cow blind diagnostic cohort by changing one choice at a time while holding the trained DAT-YOLO26 pose and segmentation branches fixed (Table 13).

Three findings are noteworthy. First, intra-mask maximum pooling outperforms 95th-percentile pooling by 2.26 percentage points (87.50% vs. 85.24%), confirming our choice of the maximum as the regional temperature estimator: although the two estimators differ by <0.15 °C per region per frame at the pixel level (Section 2.4.4), the maximum preserves the focal hot spots characteristic of early subclinical inflammation, which the percentile operator attenuates. Second, per-pixel clipping at 42 °C contributes 2.00 percentage points (87.50% vs. 85.50%): without it, occasional specular-reflection and wet-coat pixels exceeding physiologically plausible bovine-skin temperatures inflate the per-cow maximum and generate false positives. Third, replacing the trained DAT-YOLO26-pose + SVM posture gateway with a fixed threshold reduces accuracy by only 0.85 percentage points (86.65% vs. 87.50%); the learned SVM boundary therefore provides a modest but real improvement over raw keypoint geometry, most of the posture-gating benefit being attributable to the geometric cue itself. A systematic sweep of the deformable-attention insertion point across backbone stages was not performed; the present placement (Section 2.3.3) is retained throughout, and the stage-wise sweep is identified as future work.

### 3.6. Quantitative Justification of the Posture-Constrained Acquisition Strategy

The +10.00 percentage-point accuracy improvement attributable to posture filtering (Table 9) is corroborated here at the mechanism level. For every frame passing the mask-area gate ($K_{\min}=200$ px), we computed the head yaw angle $\theta \in [0^{\circ },90^{\circ }]$ from the L_Eye, R_Eye, and Nose keypoints under the convention $\theta =90^{\circ }$ for a lateral forward-moving posture and $\theta =0^{\circ }$ for a head-turning posture, using

(28) $$\begin{array}{cc} \theta & =\arctan2\left(d_{\mathrm{nose}},\;d_{\mathrm{eyes}}\right)\cdot \frac{180}{\pi }\in \left[0^{\circ },\;90^{\circ }\right]\\ d_{\mathrm{eyes}} & =∥p_{R\_Eye}-p_{L\_Eye}∥,\;d_{\mathrm{nose}}=∥p_{Nose}-\frac{1}{2}\left(p_{L\_Eye}+p_{R\_Eye}\right)∥ \end{array}$$

**Figure 9:**
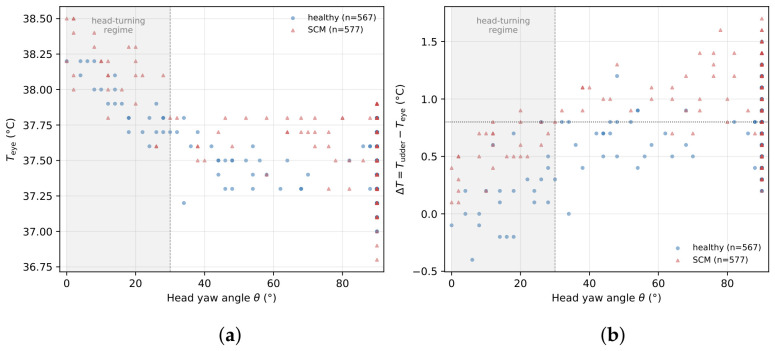
Quantitative justification of the posture-constrained acquisition strategy. Per-frame head yaw angle θ versus (**a**) the intra-mask maximum ocular temperature $T_{\mathrm{eye}}$ and (**b**) the eye–udder differential $\Delta T=T_{\mathrm{udder}}-T_{\mathrm{eye}}$, on all $N_{0}=1144$ frames from the 40-cow blind diagnostic cohort passing the mask-area gate. Shaded region: head-turning regime ($\theta <30^{\circ }$); dotted horizontal line in (**b**): the ΔT=0.8 °C decision threshold. In the head-turning regime $T_{\mathrm{eye}}$ is systematically elevated, compressing ΔT and inflating the false-negative rate among SCM cows.

Figure 9 reports θ versus $T_{\mathrm{eye}}$ and versus ΔT on all $N_{0}=1144$ frames in the 40-cow blind diagnostic cohort that passed the mask-area gate ($K_{\min}=200$ px for both the ocular and the udder mask; here “pre-filter” refers to the absence of the posture gate only, the mask-area gate having already been applied): in the head-turning regime ($\theta <30^{\circ }$) the apparent $T_{\mathrm{eye}}$ is systematically elevated by approximately 0.5 °C relative to the lateral regime, biasing the per-frame ΔT downward. The Spearman rank correlation ρ(θ,ΔT) on the pre-filter universe is +0.148 ($p=4.72\times 10^{-7}$); after the DAT-YOLO26-pose + SVM gateway rejects 105 of these 1144 mask-area-passed frames (9.2%, $N_{0}=1144\to N_{1}=1039$), the residual correlation drops to +0.023 (p=0.47), confirming that the gateway absorbs essentially all of the yaw-induced bias.

**Figure 10:**
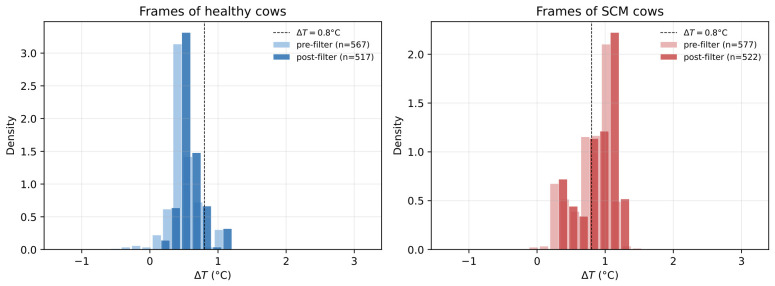
Per-cow ΔT distributions before (light) and after (dark) the DAT-YOLO26-pose + SVM posture gateway, separately for healthy (**left**) and SCM-positive (**right**) cows in the 40-cow blind diagnostic cohort. Dashed vertical line: ΔT=0.8 °C decision threshold. After posture filtering, both distributions tighten and their compressed lower tails are removed; the SCM–healthy mean separation is essentially conserved (0.321°C→0.316°C).

Figure 10 shows the per-frame ΔT distributions before and after filtering, separately for healthy and SCM cows. Three quantitative effects emerge (Table 14). First, the mean ΔT shifts upward by ≈0.02 °C for both classes (healthy: 0.644→0.665 °C; SCM: 0.965→0.981 °C), consistent with the elimination of the upward $T_{\mathrm{eye}}$ bias in head-turning frames; because this bias acts symmetrically on the two classes, the SCM–healthy mean-ΔT separation is essentially conserved across filtering (0.321 °C →0.316 °C). Second, the per-class standard deviation tightens by approximately 3–13% post-filter (healthy: 0.224→0.194 °C; SCM: 0.307→0.299 °C), reflecting the removal of geometrically biased outlier frames. Third, the long lower tails of the pre-filter distributions—frames whose ΔT has been compressed downward by $T_{\mathrm{eye}}$ inflation—are eliminated. The end-to-end +10.00 percentage-point accuracy gain reported in Table 9 therefore arises not from an expansion of the bulk SCM–healthy distance, but from the elimination of these biased boundary frames, whose effect on the diagnostic decision is amplified by the per-cow multi-frame max aggregation defined in Section 2.4.4: a single geometrically compressed frame on a SCM cow can pull the per-cow ΔT below the 0.8 °C decision threshold (boundary false negative), irrespective of the location of the bulk class distribution.

In terms of deployability, the posture gateway rejected only 105 of the 1144 mask-area-passed frames (a 9.2% posture-gate rejection rate), leaving every cow with at least $N_{\min}=5$ valid frames for a per-cow ΔT. On the NVIDIA A100 used for the diagnostic experiments the pipeline processed frames at the full 25 fps camera rate, so the latency between a cow entering the passage and the acquisition of its first valid frame is negligible relative to the in-passage transit time and does not constitute a throughput bottleneck under the present setup. We caution, however, that this figure reflects a high-end GPU; inference speed, latency, and throughput on resource-constrained edge devices in a real deployment setting remain to be evaluated (Section 5).

### 3.7. Model Interpretability and Failure-Case Analysis

**Figure 11:**
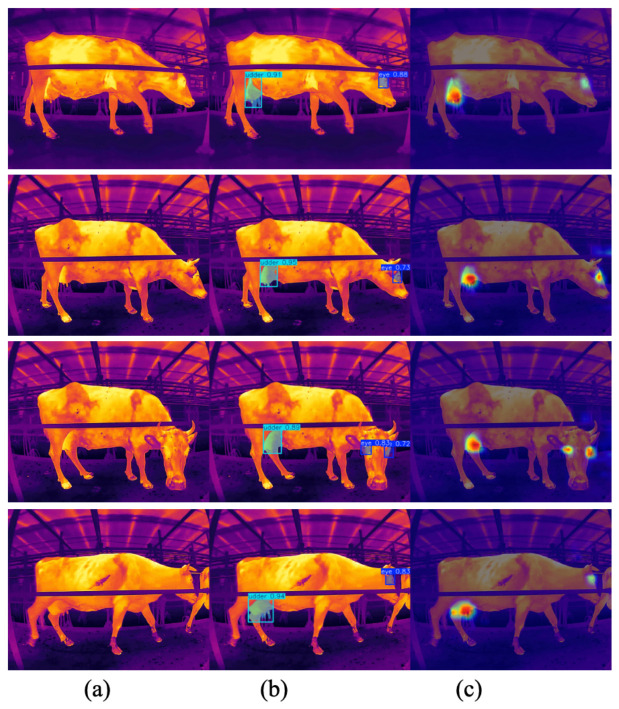
Interpretability of DAT-YOLO26-seg on four representative frames from the 40-cow blind diagnostic cohort, each row showing one frame from a different cow. (**a**) Raw infrared thermogram (pseudo-color, iron palette); (**b**) predicted instance-segmentation masks for the ocular region (blue) and the udder region (cyan), overlaid on the thermogram; (**c**) Grad-CAM activation map from the final convolutional stage of DAT-YOLO26-seg, overlaid on the thermogram (red = high gradient-weighted activation). The activation concentrates on the ocular canthus and the mammary contour and is suppressed over background structures (barn rails, ceiling, floor), confirming that the network bases its predictions on anatomically meaningful regions rather than on background thermal noise.

A central claim of this work is that the deformable attention (DAT) module enables the network to focus on critical anatomical regions—the ocular canthus and the mammary contour—while suppressing background thermal noise from barn structures such as rails, ceiling, and floor. To verify it directly, we generated Grad-CAM activation maps from the final convolutional stage of DAT-YOLO26-seg and overlaid them, together with the predicted instance-segmentation masks, on the corresponding raw thermograms (Figure 11). Grad-CAM weights each feature-map channel by the gradient of the target output with respect to that channel, so the resulting activation localizes the image regions on which the model’s prediction actually depends; if the DAT module behaves as claimed, the activation should concentrate on the ocular and mammary anatomy and fall away over the thermal background. To characterize the limits of the proposed pipeline, we examined all five misclassified cows on the 40-cow blind diagnostic cohort (two false positives, three false negatives; see the confusion matrix in Table 10).

#### 3.7.1. False Negatives (SCM Cows Scored Healthy)

The three false negatives all lay just below the decision threshold: cow ID:7982 (ΔT=0.5 °C, 0.3 °C below threshold), cow ID:1272 (ΔT=0.7 °C) and cow ID:5123 (ΔT=0.4 °C). These are boundary errors rather than gross failures: each cow carried a genuine but mild udder–eye differential consistent with early-stage subclinical infection whose thermal signature had not yet exceeded the literature-derived 0.8 °C cut-off. The Grad-CAM overlays confirm that segmentation and region attention were anatomically correct in these cases, indicating that the error originates from the intrinsic overlap between mild-SCM and healthy ΔT distributions (Figure 10) rather than from a localization or posture-gating failure. Such cases would be recoverable by a cohort-specific threshold or by the per-quarter analysis.

#### 3.7.2. False Positives (Healthy Cows Scored Mastitic)

The two false positives lay just above the threshold: cow ID:1156 (ΔT=0.9 °C, 0.1 °C above threshold) and cow ID:22131 (ΔT=1.1 °C). In both cases, the elevated ΔT is traceable to a transient, localised udder hot spot (e.g., unilateral teat-base warming from recent lying contact or residual surface moisture) rather than to a systemic infection, as confirmed by the SCC-negative ground truth and by inspection of the thermal frames. Because the per-cow aggregation uses the maximum ΔT across valid frames (Section 2.4.4), a single anomalously warm frame is sufficient to push a healthy cow above threshold; this is the price of the high sensitivity that the max-aggregation rule is designed to provide. A more conservative aggregation (e.g., an N-frame agreement requirement where N is a predefined constant parameter) would suppress such isolated false positives, and is identified as a refinement for future work.

## 4. Discussion

The significant performance gap between the experimental and control groups (Accuracy: 87.50% vs. 77.50%) underscores that standardized posture acquisition is not merely a refinement but a prerequisite for trustworthy ΔT measurement under the present setup. In the unconstrained group, arbitrary head movements and torso twisting introduced substantial systematic thermal biases. From a physical perspective, when a cow rotates its head toward the thermal sensor, the reduction in sensor-to-object distance and the change in the incidence angle lead to an artificial elevation in the apparent temperature of the ocular region. Within our diagnostic framework, which utilizes the ocular region as a thermal baseline, such posture-induced temperature spikes narrow the recorded thermal gradient ΔT. This phenomenon frequently masks the subtle thermal elevations characteristic of subclinical mastitis, leading to “false negative” outcomes and a lower sensitivity (75.00%). By enforcing a lateral forward-moving posture through the DAT-YOLO26-pose module, we effectively eliminated these distance-dependent artifacts, resulting in a relative gain of 13.33% in sensitivity.

The superiority of DAT-YOLO26 over previous models (YOLOv8–YOLO12) is primarily attributed to the integration of the Deformable Attention Transformer (DAT). Thermal images of livestock often suffer from low contrast and “thermal blur” at tissue boundaries due to heat dissipation. Traditional CNNs with fixed receptive fields struggle to delineate precise anatomical regions under these conditions. The DAT module enables the network to dynamically shift its receptive field and adaptively focus on high-contrast anatomical landmarks, such as the ocular canthus and mammary gland contours. This is evidenced by the high mask $mAP_{75}$ (0.852), which ensures that the extracted temperature data is “pure” and free from background thermal noise (e.g., barn walls or bedding). Furthermore, by adopting an NMS-free end-to-end architecture, the model has the additional advantage of avoiding mis-suppression artifacts that may occur if multiple cows enter the field of view simultaneously. While such crowded scenarios were not the focus of the present evaluation, this property may be beneficial in future studies involving free-flow barn deployment. The pose and segmentation gains reported on the frame-level inner test should be interpreted as development-stage model-selection metrics rather than definitive estimates of cross-animal generalization.

In the present cohort, the 90.00% specificity (95% Wilson CI 69.9–97.2%) is suggestive of value in minimizing false alarms, motivating larger multi-farm studies to assess whether high specificity can be sustained at scale. The framework’s lightweight design (21.8 M and 23.9 M parameters for the pose and segmentation branches, respectively) is in principle compatible with future deployment on edge computing devices in dairy barns; however, edge-side latency, throughput and accuracy were not benchmarked in the present study, and such deployment is therefore identified as future work (Section 5).

Table 15 provides an indirect comparison of the proposed framework against other representative IRT-based mastitis detection methods. Three caveats apply to the interpretation of the table. First, the test cohorts span more than an order of magnitude in size (from 22 to 160 cows), and the diagnostic-accuracy estimates from smaller cohorts (the present study and Silva et al. [17]) carry confidence intervals of approximately 25–30 percentage points (Table 10), so apparent differences of a few percent between methods cannot be interpreted as statistically meaningful. Second, the ground-truth standards differ: SCC is used in this work and in [12,13,14,15,16,18,20], while Ref. [17] relies on the California Mastitis Test, whose subjective grading can shift the operating point relative to SCC-based labels. Third, the methods belong to two distinct paradigms (Section 1): Refs. [12,13,14,15,16] and the present work report explicit eye–udder ΔT values together with an interpretable threshold, whereas Refs. [17,18,19,20,21] train networks to predict disease labels directly. Within these limits, our 87.50% accuracy is comparable to other differential-temperature methods applied to subclinical mastitis ([13]: 86.67%; [15]: 87.62%), while the higher headline accuracies of the image-classification works ([20]: 91.88%; [17]: 92.10%) are achieved on cohorts of substantially different scale and under a different output format that does not expose temperature values. Ref. [21] obtained an accuracy of 94.67%, yet it uses a small visible light dataset with unspecified cow numbers, so no direct comparison can be made with our study. The distinctive empirical contribution of the present work is not a higher point-accuracy but rather (i) the explicit quantification of the posture-screening ablation (Table 9; +10.00 percentage points accuracy and +10.26 percentage points F1 with vs. without the DAT-YOLO26-pose gate) and (ii) the preservation of an auditable ΔT output in conjunction with that ablation gain.

## 5. Limitations and Future Work

Several constraints bound the interpretation of the present results and should be read alongside the conclusions above. In particular, we highlight four principal limitations: (i) all data were collected on a single commercial farm during a single season; (ii) the independent diagnostic cohort is small (n=40 cows, 20 per class); (iii) the diagnostic ground truth is cow-level composite-milk SCC rather than bacteriological culture or quarter-level diagnosis, which may introduce some misclassification; and (iv) the framework has not yet undergone external, multi-farm validation. These points are expanded below, together with additional methodological caveats and a concrete external-validation roadmap.

First, the headline diagnostic figures are based on an independent blind cohort of n=40 cows (20 SCC^+^ and 20 SCC^−^). With this sample size, the 95% Wilson score intervals for sensitivity [64.0%, 94.8%], specificity [69.9%, 97.2%], and overall accuracy [73.9%, 94.5%] span approximately 25–30 percentage points (Table 10), indicating that the present evidence supports a preliminary, hypothesis-generating interpretation rather than a full clinical validation.

Second, the inner 7:2:1 split of the 200 development cows was performed at the frame level (Section 2.2), not at the cow level. While the final diagnostic figures rest on the cow-level 40-cow blind cohort and are therefore not subject to inter-frame leakage, the intermediate detection, keypoint and segmentation metrics reported during model development may carry a small upward bias due to inter-frame correlation. A fully cow-level inner-split protocol with retraining is identified as a near-term follow-up.

Third, acquisition-side variables that are known to influence udder surface temperature were only partially controlled. While the milking-relative timing was approximately uniform across cows (09:00–11:00 window, several hours into the inter-milking interval), the absolute degree of udder engorgement still depends on individual milk yield, parity, lactation stage, and quarter health, and was not recorded per cow. Spontaneous variation in pre-imaging activity was likewise not recorded. Frames with visibly soiled or wet udders were excluded during image-quality filtering (Section 2.1.2), but sub-visible moisture variation could remain. The wide Wilson confidence intervals reported above can therefore be interpreted as absorbing both the statistical effect of cohort size and the residual variance contributed by these uncontrolled acquisition-side factors.

Fourth, the diagnostic ground truth used in this study is composite-milk somatic cell count (SCC) at the IDF SCM threshold (>$2\times 10^{5}$ cells/mL). Composite SCC is a useful and widely used screening indicator for mastitis, but it is an indirect, cow-level proxy for udder health rather than a definitive reference such as bacteriological culture or quarter-level diagnosis; SCC values can be modulated by parity, lactation stage, recent calving, transport or heat stress, and other physiological factors, and a single-quarter infection can be diluted within a composite-sample SCC. This SCC-based classification may therefore introduce some misclassification of individual cows, and the reported metrics should be interpreted as performance against an imperfect reference standard.

Fifth, the diagnostic cohort consists exclusively of SCC-defined SCM cases and SCC-negative controls; no clinical-mastitis (CM) cases were enrolled. The current method should therefore be interpreted as a preliminary screening tool for subclinical mastitis rather than as a CM diagnostic system. Extension to three-class severity grading (healthy/SCM/CM) will require a multi-site cohort with deliberate enrollment of CM cases.

Sixth, the camera was used under its factory-calibrated radiometric mode; no in-field blackbody recalibration was performed and per-cow emissivity was not measured. This limits the absolute accuracy of reported temperatures to the factory-spec NETD (≤40 mK) plus the residual emissivity mismatch (±0.1 °C across ε∈0.96–0.98). The relative quantity ΔT on which the diagnosis rests is by construction insensitive to common-mode offsets.

Seventh, all inference timings reported in this paper were measured on a single NVIDIA A100 GPU. Edge-side latency, throughput and power consumption on resource-constrained devices (e.g., NVIDIA Jetson Orin Nano, Jetson AGX Orin) have not been measured; reported parameter counts and FLOPs should be interpreted as architectural indicators, not as performance guarantees on edge hardware. Dedicated edge-deployment benchmarking is identified as the natural next engineering step.

A specific refinement concerns the per-cow temperature aggregation rule. The present pipeline assigns each cow the maximum ΔT across all valid frames (Algorithm 1), which maximizes sensitivity to the focal hot spots characteristic of early subclinical inflammation but can, in principle, be driven by a single spuriously warm frame arising from specular reflection or a wet-surface artifact—the mechanism underlying the boundary false positives discussed in Section 3.7. In future work we will replace the single-frame maximum with a multi-frame-corroborated maximum: the peak ocular and udder temperatures will be accepted only when reproduced, within a tolerance band, across at least *m* of the valid frames (a multi-frame agreement criterion), or computed as the maximum of a short temporal moving average rather than of the raw per-frame values. This is expected to preserve the sensitivity of maximum-pixel pooling to genuine focal warming while suppressing transient single-frame, single-pixel artifacts. It is identified as part of the planned multi-site validation.

### External-Validation Roadmap

All data in the present study were collected from a single commercial dairy farm in Hohhot, Inner Mongolia, China, during a single season (May 2025). Environmental factors (ambient temperature, humidity, ventilation, barn architecture, photoperiod), management practices (milking schedule, hygiene protocol, bedding type), animal-level factors (genetic background, body condition, parity distribution, lactation-stage mix), and climatic factors can all influence the absolute and relative thermal signatures of the ocular and mammary regions. The generalizability of the reported figures to other farms, regions, breeds, and climatic conditions therefore remains untested, and the framework should not be considered ready for widespread deployment without prospective multi-site validation. Three concrete validation steps are identified: (1) multi-farm replication within Inner Mongolia (2–3 additional commercial farms; breed and climate held approximately constant); (2) multi-region/multi-climate validation in subtropical and temperate-maritime regions; (3) multi-breed validation paired with the bacteriological reference upgrade described above.

## 6. Conclusions

This study developed and preliminarily evaluated a two-stage posture-constrained infrared thermography (IRT) framework based on the DAT-YOLO26 model for the automated detection of dairy cow mastitis. By integrating deep learning-based pose estimation with high-precision instance segmentation, the framework addresses a critical gap in precision livestock farming: the systematic measurement bias caused by uncontrolled animal movement.

The integration of the DAT-YOLO26-pose module as a quality gateway proved to be the decisive factor in measurement consistency. Experimental results demonstrated that enforcing a lateral forward-moving posture increased diagnostic accuracy from 77.50% to 87.50%. From a physical standpoint, the study confirms that head rotation toward the sensor induces an artificial elevation in apparent ocular temperature due to reduced sensor-to-object distance. Within a differential diagnostic framework, this posture-induced baseline shift narrows the thermal gradient between the eye and the udder, frequently leading to “false negative” outcomes. By mitigating these geometric artifacts, the proposed pipeline enhanced diagnostic sensitivity by 10 percentage points (a relative gain of 13.33%).

Furthermore, the architectural innovation of the Deformable Attention Transformer (DAT) enabled the model to overcome the inherent challenges of “thermal blur” and low contrast in infrared imagery. Achieving a Mask $mAP_{75}$ of 0.852, the system supports high-fidelity anatomical delineation, which is essential for accurate temperature extraction. With a high specificity of 90.00% and a lightweight computational profile, the framework shows promising potential for future on-farm edge deployment, with comprehensive benchmarking planned in subsequent work.

The results reported in this paper constitute a preliminary, single-farm evaluation of the posture-constrained framework. Generalizability across farms, breeds, climates, seasons, and management practices remains untested; multi-farm prospective validation, ideally coupled with quarter-level bacteriological reference, is the necessary next step before practical deployment can be recommended.

In summary, this research provides a non-invasive screening approach that may help reduce the risk of undetected subclinical mastitis, pending multi-farm validation. The findings emphasize that identifying the ideal measurement posture is a fundamental prerequisite for transitioning infrared diagnostics from controlled laboratory settings to autonomous, high-throughput dairy farm applications.

## Figures and Tables

**Table 1:** Data partition summary. The outer split (40-cow blind diagnostic cohort vs. 200 development cows) is cow-level disjoint; the inner 7:2:1 split of the 200 development cows is frame-level. The diagnostic figures reported in Section 3.5 rest on the cow-level blind cohort and are therefore not subject to inter-frame leakage.

Subset	Purpose	Partition Level	Number of Images
Training	Detector/pose/seg. fitting	frame-level (within 200 dev. cows)	∼4900
Validation	Hyper-parameter/early stopping	frame-level (within 200 dev. cows)	∼1400
Inner test	Architecture comparison; mAP, keypoint, mask metrics	frame-level (within 200 dev. cows)	∼700
Blind diagnostic cohort	End-to-end diagnostic accuracy (Section 3.5)	cow-level (a priori reserved 40 cows)	∼1200

**Table 2:** Definition and anatomical description of the simplified cow keypoints used for pose estimation.

ID	Keypoint	Description	ID	Keypoint	Description
1	L_Eye	Left eye canthus	6	TailBase	Coccygeal vertebrae base
2	R_Eye	Right eye canthus	7	L_F_Paw	Left front hoof
3	Nose	Muzzle center	8	R_F_Paw	Right front hoof
4	Throat	Ventral neck area	9	L_B_Paw	Left back hoof
5	Withers	Interscapular region	10	R_B_Paw	Right back hoof

**Table 3:** Labeling specifications for the instance segmentation task.

Class Label	Target Region	Annotation Scope
eye	Ocular Region	Orbital area including the eyeball and eyelid
udder	Udder Region	Entire udder surface including the four quarters

**Table 4:** Experimental Hardware and Software Environment.

Category	Configuration
CPU	Intel(R) Xeon(R) Gold 5218 CPU @ 2.30 GHz
GPU	NVIDIA A100 80GB PCIe
RAM	256 GB DDR4
Operating System	Ubuntu 24.04.1 LTS
Programming Language	Python 3.10
Deep Learning Framework	PyTorch 2.6.0
CUDA	12.8
OpenCV	4.11.0
Annotation Tool	X-AnyLabeling 3.0

**Table 5:** Quantitative comparison of pose estimation performance across different YOLO architectures.

Model	${\mathrm{mAP}}_{50}$	${\mathrm{mAP}}_{75}$	${\mathrm{mAP}}_{50:95}$	Params (M)	GFLOPs
YOLOv8m-pose	0.882	0.745	0.723	26.4	81.0
YOLOv9m-pose	0.896	0.752	0.711	32.1	121.1
YOLOv10m-pose	0.902	0.766	0.745	24.4	120.3
YOLO11m-pose	0.895	0.768	0.755	20.9	71.4
YOLO12m-pose	0.909	0.784	0.775	20.2	67.5
YOLO26m-pose (Baseline)	0.923	0.824	0.796	21.5	73.1
DAT-YOLO26m-pose	0.935	0.852	0.814	21.8	73.5

**Table 6:** Performance comparison of posture classification algorithms.

Classification Method	Accuracy (%)	Precision (%)	Recall (%)	F1-Score (%)
Threshold-Based Method	88.57	87.23	89.00	88.11
SVM-Based Method	94.29	93.88	94.50	94.19

**Table 7:** Quantitative comparison of instance segmentation performance across different YOLO architectures.

Model	${\mathrm{mAP}}_{50}$	${\mathrm{mAP}}_{75}$	${\mathrm{mAP}}_{50:95}$	Params (M)	GFLOPs
YOLOv8m-seg	0.902	0.775	0.743	27.2	110.2
YOLOv9m-seg	0.915	0.782	0.741	35.8	158.4
YOLOv10m-seg	0.922	0.806	0.765	26.5	140.3
YOLO11m-seg	0.925	0.818	0.782	22.4	104.5
YOLO12m-seg	0.934	0.834	0.805	21.6	98.6
YOLO26m-seg (Baseline)	0.942	0.843	0.816	23.6	102.3
DAT-YOLO26m-seg	0.956	0.882	0.852	23.9	102.7

**Table 8:** Stability of the architectural improvement across three independent training runs (random seeds s∈{default,42,124}, evaluated on the inner-test subset). Values are mean ± sample standard deviation (n=3); Δ is the mean DAT-YOLO26 advantage; *p* is from a two-sided paired *t*-test on the three seed-matched runs. The inner-test subset is frame-level (Section 2.2), so these metrics are used for architecture comparison and not as diagnostic claims.

Metric	YOLO26	DAT-YOLO26	Δ	Paired *p*
Pose estimation				
mAP_50_	0.920±0.006	0.934±0.001	+0.014	0.044
mAP_75_	0.823±0.001	0.854±0.002	+0.031	0.002
mAP_50:95_	0.792±0.005	0.815±0.001	+0.023	0.019
Instance segmentation				
mAP_50_	0.943±0.002	0.955±0.001	+0.012	0.018
mAP_75_	0.844±0.002	0.882±0.001	+0.037	0.001
mAP_50:95_	0.816±0.002	0.852±0.001	+0.037	0.0003

**Table 9:** Diagnostic performance comparison with/without posture recognition screening.

Experimental Group	Accuracy (%)	Precision (%)	Recall (Sensitivity) (%)	Specificity (%)	F1-Score (%)
YOLO26 Without Posture	70.00	72.22	65.00	75.00	68.42
YOLO26 With Posture	75.00	77.78	70.00	80.00	73.68
DAT-YOLO26 Without Posture	77.50	78.94	75.00	80.00	76.92
DAT-YOLO26 With Posture	87.50	89.47	85.00	90.00	87.18

**Table 10:** Confusion matrix and per-metric 95% Wilson score confidence intervals of the complete posture-constrained pipeline on the 40-cow blind diagnostic cohort (20 healthy, 20 mastitis-positive; ground truth: composite-milk somatic cell count with the IDF threshold $\mathrm{SCC}>2\times 10^{5}$ cells/mL). The intervals are deliberately reported on a sample size of n=20 per class so that the precision attainable with the present cohort is empirically transparent; multi-farm validation on larger cohorts is identified as future work (Section 5).

Item	Actual Mastitis (SCC^+^)	Actual Healthy (SCC^−^)	Total
Predicted mastitis	TP =17	FP =2	19
Predicted healthy	FN =3	TN =18	21
Total	20	20	40
**Metric**	**Point estimate**	**95% Wilson CI**	
Accuracy	87.50%	[73.89%, 94.54%]	
Sensitivity (recall)	85.00%	[63.96%, 94.76%]	
Specificity	90.00%	[69.90%, 97.21%]	
PPV (precision)	89.47%	[68.61%, 97.06%]	
NPV	85.71%	[65.36%, 95.02%]	
F1-score	87.18%	—	

**Table 11:** Paired agreement between the proposed DAT-YOLO26 + posture-filter pipeline and the baseline YOLO26 configuration on the 40-cow blind diagnostic cohort, used for McNemar’s exact test. Each cell counts cows.

		Baseline YOLO26	
		Correct	Incorrect
DAT-YOLO26 with Posture	correct	28	7
	incorrect	0	5

**Table 12:** Class-conditional temperature statistics on the 40-cow blind diagnostic cohort (mean ± SD, °C) and Mann–Whitney *U* tests between SCC^+^ and SCC^−^ cows.

Variable	Healthy (n=20)	SCM (n=20)	*U*	*p*
$T_{\mathrm{eye}}$	37.53±0.12	37.53±0.17	199.0	0.99
$T_{\mathrm{udder}}$	38.18±0.18	38.52±0.30	68.5	$3.6\times 10^{-4}$
ΔT	0.65±0.15	0.98±0.27	69.0	$3.9\times 10^{-4}$

**Table 13:** Extended ablations on the 40-cow blind diagnostic cohort. Each variant changes one design choice while holding the trained DAT-YOLO26 pose and segmentation branches fixed; the default configuration (maximum pooling, 42 °C clipping, DAT-YOLO26-pose + SVM gateway) attains 87.50% accuracy. ΔAcc. is relative to the default.

Ablation Axis	Variant	Acc. (%)	ΔAcc. (pp)
Temperature pooling	Maximum (default)	87.50	—
	95th percentile	85.24	−2.26
Pixel clipping at 42 °C	With clipping (default)	87.50	—
	Without clipping	85.50	−2.00
Posture gateway	DAT-YOLO26-pose + SVM (default)	87.50	—
	Keypoint threshold	86.65	−0.85

**Table 14:** Posture-filter throughput on the 40-cow blind diagnostic cohort. Pre-filter universe = all frames passing the mask-area gate ($K_{\min}=200$ px on both the ocular and udder masks); post-filter universe = additionally classified as lateral forward-moving by DAT-YOLO26-pose + SVM. “Pre-filter” refers to the absence of the posture gate only. Spearman ρ is computed between the head yaw angle θ and ΔT.

Statistic	Pre-Filter	Post-Filter
Number of frames	1144	1039
Posture-gate rejection rate	—	9.2%
Mean ΔT (healthy) [°C]	+0.64±0.22	+0.67±0.19
Mean ΔT (SCM) [°C]	+0.97±0.31	+0.98±0.30
Mean separation (SCM − healthy) [°C]	+0.321	+0.316
Spearman ρ(θ,ΔT)	+0.148	+0.023
*p*-value	$4.72\times 10^{-7}$	0.47

**Table 15:** Indirect comparison with representative IRT-based dairy cow mastitis detection methods. Because the cited studies differ substantially in test-cohort size, ground-truth standard (SCC, CMT, or experimental challenge), label granularity (binary vs. three-class severity), and methodological paradigm (eye–udder differential-temperature vs. end-to-end image classification, cf. Section 1), the numbers below should be interpreted as indirect literature reference values rather than as results of a head-to-head benchmark on a common dataset; we report all values as stated in the original publications. Our point estimates are accompanied by 95% Wilson confidence intervals (Table 10); confidence intervals for prior works are reported here only when given in the cited source.

Study (Ref.)	Approach	Dataset (Number of Imgs/Number of Cows)	Test Cohort	Ground Truth	Acc. (%)	Sens. (%)	Spec. (%)	F1 (%)
Wang et al. 2021 [12]	Improved YOLOv3-tiny for eye/udder ROI; threshold on ΔT	1110/—	22 (12 N + 10 M)	SCC	77.30	—	—	—
Zhang et al. 2020 [16]	EFMYOLOv3 + eye/udder ROI; threshold on ΔT	6000/300	30 (17 N + 13 M)	SCC	83.33	92.31	76.47	—
Wang et al. 2022 [15]	YOLOv5 + bilateral $T_{\mathrm{udder}}$ + $T_{\mathrm{eye}}$ differential	3000/198	105 (78 N + 27 M)	SCC	87.62	96.30	84.62	—
Zhang et al. 2023 [13]	CLE-UNet (ECA + centroid loss) segmentation; ΔT rule	2400/180	30 (15 N + 15 M)	SCC	86.67	82.35	80.00	87.50
Wang et al. 2024 [14]	DeepLabV3 + and improved YOLOv5; ΔT rule	1213/200	50 (34 N + 10 SCM + 6 CM)	SCC	86.00	79.41	92.49	—
Chu et al. 2023 [18]	YOLOv7 + CenterNet keypoints; SVM-wrapper fusion of temperature and udder size features	3565/196	79 (55 N +16 SCM + 8 CM)	SCC	88.61 ^†^	81.25 ^††^	91.94 ^††^	—
Chu et al. 2025 [19]	MS-scSE-DenseNet-201; segmentation + motion deblurring; three-class severity	5000/802	160 (115 N + 25 SCM + 20 CM)	SCC	90.18	95.45	84.00	—
Chu et al. 2025 [20]	Multi-feature image layers fusion; DenseNet-201; three-class severity	7000/802	80 (35 N + 25 SCM + 20 CM)	SCC	91.88	84.00	90.57	—
Silva et al. 2024 [17]	ResNet50 + sequential cross-domain transfer learning; Bayesian optimization	165/55	42 (21 N + 21 M)	CMT	92.10	—	—	90.00
Sharvanthika et al. 2024 [21]	ResNet50 + CNN + ViT hybrid framework + data augmentation; Visible light udder images	200/–	—	—	94.67	—	—	—
This work	DAT-YOLO26-pose + DAT-YOLO26-seg + eye–udder ΔT rule	7000/200	40 (20 N + 20 M)	SCC	87.50 [73.9, 94.5]	85.00 [64.0, 94.8]	90.00 [69.9, 97.2]	87.18

^†^ Overall (binary) accuracy on 79 cows; ^††^ subclinical-mastitis (SCM) sensitivity and specificity; clinical-mastitis (CM) values: 87.50%/94.03%. 95% Wilson CIs in square brackets; N stands for normal samples, and M represents mastitis-positive samples; dashes (—) = not reported.

## Data Availability

The dataset supporting the findings of this work is available from the corresponding author on request. Public release of the data is restricted due to ongoing follow-up research.
